# Gain of 1q confers an MDM4-driven growth advantage to undifferentiated and differentiating hESC while altering their differentiation capacity

**DOI:** 10.1038/s41419-024-07236-x

**Published:** 2024-11-21

**Authors:** Nuša Krivec, Edouard Couvreu de Deckersberg, Yingnan Lei, Diana Al Delbany, Marius Regin, Stefaan Verhulst, Leo A. van Grunsven, Karen Sermon, Claudia Spits

**Affiliations:** 1 Research Group Genetics, Reproduction and Development, Faculty of Medicine and Pharmacy, Vrije Universiteit Brussel, 1090 Brussels, Belgium; 2 Liver Cell Biology Research Group, Faculty of Medicine and Pharmacy, Vrije Universiteit Brussel, 1090 Brussels, Belgium

**Keywords:** Cell death, Embryonic stem cells, Differentiation

## Abstract

Gain of 1q is a highly recurrent chromosomal abnormality in human pluripotent stem cells. In this work, we show that gains of 1q impact the differentiation capacity to derivates of the three germ layers, leading to mis-specification to cranial placode and non-neural ectoderm during neuroectoderm differentiation. Also, we found a weaker expression of lineage-specific markers in hepatoblasts and cardiac progenitors. Competition assays show that the cells retain their selective advantage during differentiation, which is mediated by a higher expression of *MDM4*, a gene located in the common region of gain. *MDM4* drives the winner phenotype of the mutant cells in both the undifferentiated and differentiating state by reducing the cells’ sensitivity to DNA damage through decreased p53-mediated apoptosis. Finally, we found that cell density in culture plays a key role in promoting the competitive advantage of the cells by increasing DNA damage.

## Introduction

At present, over 7400 human pluripotent stem cell (hPSC) lines have been registered at the hPSC lines registry (https://hpscreg.eu), and over 50 ongoing and completed clinical trials involve the transplantation of cells derived from hPSC [1, 2]. While the ability to differentiate is a fundamental characteristic of all hPSCs, they may differ in their differentiation capacity to specific lineages or cell types [3]. Causes for this variability are common genetic variation [4, 5], epigenetic variance [6–8], mitochondrial mutations [9, 10] and a broad range of environmental factors induced by differences in culture conditions [3]. Another significant source of variation is the genetic changes acquired by cells during in vitro culture, such as copy number variations [11, 12] and single nucleotide changes, including p53-inactivating mutations [13–15].

Full or segmental gains of chromosomes 1, 12, 17 and 20 are known to be highly recurrent in both human induced pluripotent stem cells (hiPSC) and human embryonic stem cells (hESC) [11, 12, 16]. They confer increased cloning efficiency, shorter doubling times, decreased sensitivity to apoptotic triggers, and increased self-renewal and growth factor independence to the cells [17–21]. This results in a growth advantage in the undifferentiated state and a rapid culture takeover by the mutant cells [19, 22]. Presently, the mechanisms and driver gene behind this selective advantage have been fully characterized for only one of these recurrent abnormalities: the gain of 20q11.21 results in a decreased sensitivity to apoptosis due to higher *BCL2L1* expression [18, 19]. Whether this selective advantage is maintained during differentiation remains unanswered. Further, there is increasing evidence that these recurrent abnormalities affect the differentiation capacity of hPSC, often in a cell-lineage-specific manner [17, 20, 23]. Gains of 20q11.21 and losses of 18q lead to impaired neuroectoderm commitment [24–26], and gain in chromosome 12p results in large foci of residual undifferentiated cells persisting after hepatoblast differentiation [27].

In this study, we focused on the gain of 1q, which is one of the most commonly acquired genetic abnormalities in hPSC worldwide [11]. Despite that, little is known about its functional effects. Human ESC with a complex karyotype, including a gain of 1q, have an altered gene expression profile with active WNT signaling and deregulation of tumor suppressors and oncogenes, as well as differentiation bias towards ectoderm [23]. Gain of 1q was also found to recurrently appear during in vitro neural differentiation, suggesting a selective advantage during differentiation [28]. In this work, we found that gain of 1q alters hESC differentiation and that *MDM4*, a p53 regulator within the minimal region of gain, drives competitive advantage by reducing sensitivity to DNA damage-induced p53-mediated apoptosis in both undifferentiated and differentiating states.

## Results

### hESC^1q^ mis-specify to alternative cell fates during neuroectoderm differentiation and differentiate to more immature hepatoblasts and cardiac progenitors than hESC^wt^

**Fig. 1:**
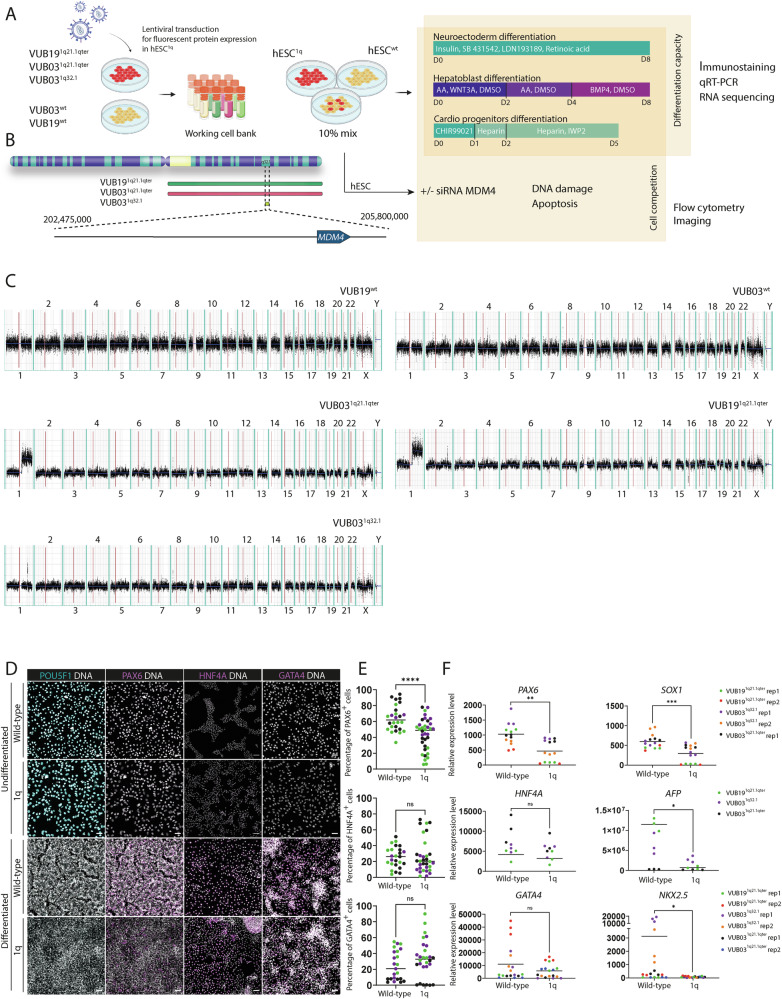
hESC lines with a gain of 1q show impaired neuroectoderm differentiation. **A** Lentiviral transduction was used to induce stable expression of fluorescent proteins in hESC^1q^. Working cell banks were established comprising mutant-labeled cell lines and their isogenic counterparts. Pure wild-type and 1q lines were utilized, along with mixes containing 10% of 1q cells, for differentiation to three lineages: neuroectoderm, hepatoblasts and cardiac progenitors. Differentiation capacity was assessed through immunostaining, qPCR, and RNA sequencing. Cell competition results were measured by analyzing the ratio of mutant and wild-type cells before and after differentiation using flow cytometry and immunostaining. To study the role of *MDM4* in the growth advantage of hESC^1q^, *MDM4* was downregulated by siRNA prior to the competition assays. To study the mechanisms by which *MDM4* provides the selective advantage, DNA damage was induced by Bleomycin, and apoptosis levels were evaluated using Annexin V staining and flow cytometry, while DNA damage levels were studied through immunostaining for gamma-H2AX. **B** Breakpoints for the minimal region of gain in chromosome 1 are 202,475,000 and 205,800,000. **C** Shallow DNA sequencing–based karyotyping of the hESC lines included in the study. Gain in 1q chromosome is visible for VUB03^1q32.1^, VUB03^1q21.1qter^ and VUB19^1q21.1qter^. **D** Representative images of immunostainings for POU5F1 (turquoise, 1st panel), PAX6 (magenta, 2nd panel), HNF4A (magenta, 3rd panel) and GATA4 (magenta 4th panel) of hESC^wt^ and hESC^1q^ before and after the completion of induction of differentiation to neuroectoderm (NE, day 8), hepatoblast (HEP, day 8), cardiac progenitors (CP, day 5) of VUB19^1q21.1qter^. Figure S1 shows the images for cell lines VUB03^1q21.1qter^ and VUB03^1q32.1^ included in the study. **E** Quantification of the percentage of PAX6, HNF4A and GATA4-positive cells in the immunostainings shown in panel D and in Fig. S1. **F** mRNA quantification by quantitative real-time PCR of *PAX6* and *SOX1* in NE^wt^ and NE^1q^, *HNF4A* and *GATA4* in HEP^wt^ and HEP^1q^, and *GATA4* and *NKX2.5* in CP^wt^ and CP^1q^. Results for *POU5F1* and *NANOG* are shown in Fig. S1. Each differentiation was carried out in three technical replicates at the same time. Different hESC lines and independent experimental replicates (rep) are coded in different colors. *p < 0.05, **p < 0.01, ****p < 0.0001. ns = non-significant.

Figure 1A shows the overall setup of this study. All experiments were carried out on our in-house hESC lines VUB03 and VUB19 [29, 30]. In vitro culture led to three independent events of gain in chromosome 1 (Fig. 1B, C). VUB19 and one subline of VUB03 acquired a gain of the entire q arm of chromosome 1 (termed VUB19^1q21.1qter^ and VUB03^1q21.1qter^), another subline of VUB03 gained a smaller region spanning 3.3 Mb in 1q32.1 (VUB03^1q32.1^).

We investigated the impact of the gain of 1q on trilineage differentiation by subjecting hESC^wt^ and hESC^1q^ to neuroectoderm (NE), hepatoblast (HEP) and cardiac progenitor (CP) differentiation (VUB03^1q21.1qter^, VUB19^1q21.1qter^, VUB03^1q32.1^, VUB03^wt^ and VUB19^wt^, all lines differentiated at least in triplicate). We measured the expression of six lineage-specific markers and of *NANOG* and *POUF51* to evaluate differentiation efficiency and to test for residual undifferentiated cells (Figs. 1D–F, S1). All differentiated cells had almost undetectable levels of *POU5F1* and *NANOG* mRNA (Fig. S1), and no POU5F1-positive cells appeared in the immunostainings (Fig. 1D), indicating that all cells exited the undifferentiated state (Figs. 1D, S1).

Immunostaining showed a lower percentage of PAX6-positive cells in differentiated hESC^1q^ than in their isogenic hESC^wt^ counterparts (Figs. 1D, 1E, NE^1q^ = 43.78%, NE^wt^ = 64.19%, *p* < 0.0001, unpaired *t*-test, *N*^wt^ = 26, *N*^1q^ = 36). While the number of SOX1-positive cells was similar between the two groups, we observed a significantly reduced intensity of SOX1 protein expression in hESC^1q^ (Fig. S2A, B, NE^1q^ = 6343, NE^wt^ = 9107, *p* < 0.0001, unpaired *t*-test, *N*^wt^ = 13, *N*^1q^ = 18). In line with this, NE^1q^ showed significantly lower mRNA levels of *PAX6* and *SOX1* compared to the levels in NE^wt^ (Fig. 1F p^*PAX6*^ = 0.0017 and p^*SOX1*^ = 0.0002, unpaired *t*-test, *N* = 15), indicating a decreased neuroectodermal differentiation efficiency. For the HEP differentiation, we found no significant difference between HEP^wt^ and HEP^1q^ in the mRNA expression levels of *HNF4A* while *AFP* had a significantly lower expression in HEP^1q^ (Fig. 1F, *p*^HNF4A^ = 0.2247, *p*^AFP^ = 0.0142, unpaired *t*-test, *N* = 9) and immunostaining for HNF4A showed similar percentages of HNF4A positive cells in both groups (Fig. 1E, HEP^1q^ = 26.27%, HEP^wt^ = 25.40%, *p* = 0.8585, unpaired *t*-test, *N*^wt^ = 25, *N*^1q^ = 32). Similarly, the mRNA levels of the CP marker *GATA4* was not significantly different between CP^wt^ and CP^1q^ while *NKX2-5* was significantly lower in CP^1q^ (Fig. 1F, *p*^GATA4^ = 0.1537, *p*^*NKX2.5*^ = 0.0398, unpaired *t*-test, *N* = 18), which was confirmed by the immunostaining for GATA4 (Fig. 1E, CP^1q^ = 33.35%, CP^wt^ = 24.74%, *p* = 0.1627, unpaired *t*-test, *N*^wt^ = 23, *N*^1q^ = 28).

Taken together, hESC^1q^ show a decreased differentiation efficiency into neuroectoderm, but do not remain undifferentiated, suggesting that part of the cells mis-specify to alternative cell fates. hESC^1q^ commit to mesendoderm as efficiently as their isogenic counterpart, showing similar early endoderm and mesoderm marker expression but reduced expression of markers for further cell-type commitment.

To further characterize the differentiated cells, we carried out bulk mRNA sequencing of 43 samples: 15 samples of neuroectoderm (NE^wt^ = 6, NE^1q^ = 9), 14 of cardiac progenitors (CP^wt^ = 6, CP^1q^ = 8) and 14 of hepatoblasts (HEP^wt^ = 6, HEP^1q^ = 8). Differential gene expression analysis showed that NE^1q^ differentially expressed 1603 genes as compared to NE^wt^, while HEP^1q^ and CP^1q^ differentially expressed 189 and 241 genes, respectively, as compared to HEP^wt^ and CP^wt^ (Fig. S3). We surmised that this significant difference in the number of differentially expressed genes was caused by mis-specification of hESC^1q^ to neuroectoderm and were rather yielding a mixed cell population, which did not occur in the mesendoderm lineages.

**Fig. 2:**
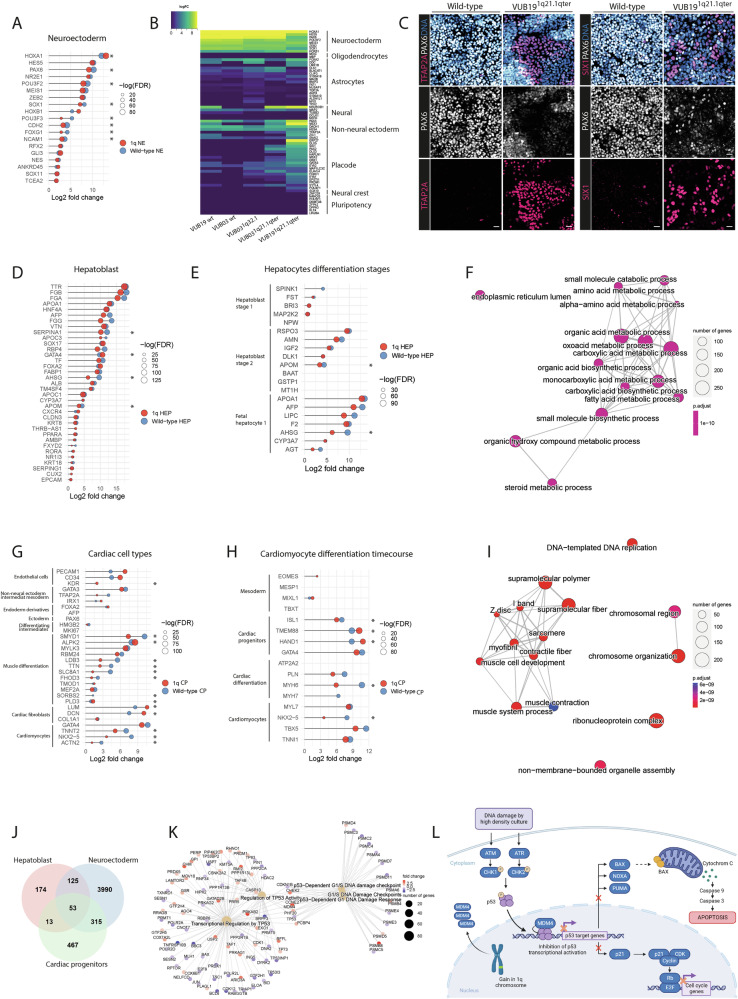
hESC^1q^ mis-specify to alternative cell fates during neuroectoderm differentiation and differentiate to more immature hepatoblasts and cardiac progenitors than hESC^wt^. **A** Lollipop diagrams representing the differential gene expression of neuroectoderm markers in NE^wt^ and NE^1q^ vs undifferentiated cells. All genes shown in this panel and in (**D**, **E**, **G** and **H**) are differentially expressed with a log_2_fold-change over 0 and FDR < 0.05. **B** Heatmap representing the induction of expression of genes marking different ectodermal cell types and the undifferentiated hESC state in NE obtained from the five lines included in this study. **C** Representative images of the immunostaining of NE^wt^ and NE^1q^ for DNA (blue), PAX6 (white), TFAP2A (red, left panel) and SIX1 (red, right panel). **D** Lollipop diagrams representing the differential gene expression of hepatoblast markers in HEP^wt^ and HEP^1q^ vs undifferentiated cells. **E** Lollipop diagrams representing the differential gene expression of markers of different stages of hepatocyte differentiation in HEP^wt^ and HEP^1q^ vs undifferentiated cells. **F** Enrichment map showing the Top-15 deregulated pathways from gene ontology gene set enrichment analysis in HEP^wt^ vs HEP^1q^. The size of nodes indicates the number of genes in each pathway and the color represents adjusted *p*-value. Pathways that cluster together have overlapping gene sets. **G** Lollipop diagrams representing the differential gene expression of genes marking different cardiac cell types in CP^wt^ and CP^1q^ vs undifferentiated cells. **H** Lollipop diagrams representing the differential gene expression of genes marking different stages of cardiomyocyte differentiation in CP^wt^ and CP^1q^ vs undifferentiated cells. **I** Enrichment map showing the top-15 deregulated pathways from gene ontology gene set enrichment analysis in CP^wt^ vs CP^1q^. **J** Venn diagram showing the overlaps in differentially expressed genes between control and 1q-cells, for the three cell types with log_2_fold-change over 0 and FDR < 0.05. *MDM4* is part of the core of 53 commonly deregulated genes. **K** Reactome pathway gene set enrichment analysis of the C2 library canonical pathways related to p53 signaling in NE, HEP and CP from 1q and wildtype cells. **L** Graphical summary of the model in which *MDM4* has a higher expression because of the gain of 1q, resulting in an inhibition of the p53 signaling pathway and in turn decreasing the sensitivity of the cells to DNA damage.

To determine the alternate cell fate acquired by hESC^1q^ upon neuroectoderm differentiation, we carried out differential gene expression analysis of the NE^1q^ and the NE^wt^ relative to bulk RNA sequencing data of undifferentiated hESC (*N* = 38 samples, previously published data [26]). First, we tested the expression of neuroectoderm markers, which we found to be less induced in NE^1q^ than in NE^wt^ (Figs. 2A, S4A). We then queried the data for the expression of different sets of markers for embryonic and extra-embryonic lineages that appear in early human development. Table S1 shows the lists of gene sets we curated from published data [31–54]. We found that VUB19^1q21.1^ showed high expression of markers of non-central nervous system (non-CNS) ectodermal lineages such as non-neural ectoderm and of cranial placode (Figs. 2B, S4B, S5). Staining for the non-CNS marker TFAP2A and placode marker SIX1 of independent differentiation experiments of VUB19^1q21.1^ confirmed that the alternate cell fate of this line was indeed consistently a mix of neuroectoderm and non-CNS cells (Fig. 2C). The other two lines showed variable expression of genes of different lineages, including the neuronal, but we could not identify a gene expression pattern consistent with a specific cell type (Figs. 2B, S4B, S5).

Next, we used the same approach to study the cell types obtained from the HEP and CP samples. While hESC^1q^ equally induce some of the early HEP and CP differentiation markers as hESC^wt^, 68.6% (24/35) and 63.2% (12/19) of HEP and CP lineage-specific markers have a lower expression in the mutant cells (Fig. 2D, G). Because the HNF4 and GATA4 staining (Figs. 1D, S1) and the RNA sequencing data (Fig. S6) did not suggest mis-specification for these lineages, we analyzed the gene expression data with the aim of establishing the degree of maturity of the HEP and CP cells.

Human ESC-derived HEP have a profile between hepatoblast stage 2 and fetal hepatocyte 1 (Fig. 2E), and cells with a gain of 1q show overall lower expression of 64% of markers (16/25). Gene-set enrichment analysis of HEP^wt^ versus HEP^1q^ showed that 145 of the 162 significantly enriched gene sets in the canonical pathways gene set had negative enrichment scores. They were frequently related to key processes of hepatocyte and liver function, including cholesterol metabolism, ferroptosis, transsulfuration, plasma lipoprotein remodeling, folate metabolism, selenium micronutrient network and metabolism of steroids (FDR < 0.05, Table S2). Gene ontology enrichment analysis showed that 1574 of the 1913 gene sets had negative enrichment scores, including amino acid metabolic process, fatty acid metabolic process and steroid metabolic process (Fig. 2F, Table S2).

In the case of CP, CP^1q^ and CP^wt^ equally express a profile between cardiac progenitors and cardiomyocytes, where CP^1q^ have lower expression of genes marking the later stages of differentiation (Fig. 2H). Gene set enrichment analysis of the CP samples showed that CP^1q^ have negative enrichment scores for 157 of the 178 significantly enriched canonical pathway gene sets, including sets related to dilated cardiomyopathy, folding of actin, striated muscle contraction and hypertrophic cardiomyopathy (Table S2). These genes are key to correct heart contraction functions. Gene ontology enrichment analysis identified negative enrichment scores for 1146 of the 1786 significantly enriched set, including terms such as l band, Z disc, sarcomere, myofibril, contractile fiber, muscle system process and muscle development (Fig. 2I, Table S2).

### Human ESC^1q^ have an *MDM4*-driven competitive advantage that is retained during differentiation

Human ESC^1q^ have a well-established competitive advantage over their genetically balanced counterparts in the undifferentiated state [11, 13, 16]. We next aimed at determining whether the cells retain this selective advantage during differentiation, and which gene is driving their winning phenotype.

We first looked at the 53 commonly deregulated genes in our HEP, CP and NE samples (Fig. 2J), and found that *MDM4*, a regulator of p53 activity that is located in the common region of gain and which has been previously suggested as a key gene for the gain of 1q [13], is consistently upregulated in all samples (Figs. 2J, S2C). Gene set enrichment analysis of the differentially expressed genes in NE, HEP and CP from 1q and wildtype cells for the Reactome pathways related to p53 signaling indeed shows that the transcriptional regulation by p53, the regulation of p53 activity, the G1/S damage checkpoint and the p53 dependent responses to DNA damage in G1 and S are all significantly negatively enriched (Figs. 2K, S2D, Tables S2, S3). This led us to the hypothesis that the higher expression of *MDM4* in cells with a gain of 1q results in the inhibition of p53-mediated transcriptional activity, leading to a decreased induction of apoptosis, thus providing a competitive advantage to the mutant cells (Fig. 2L).

**Fig. 3:**
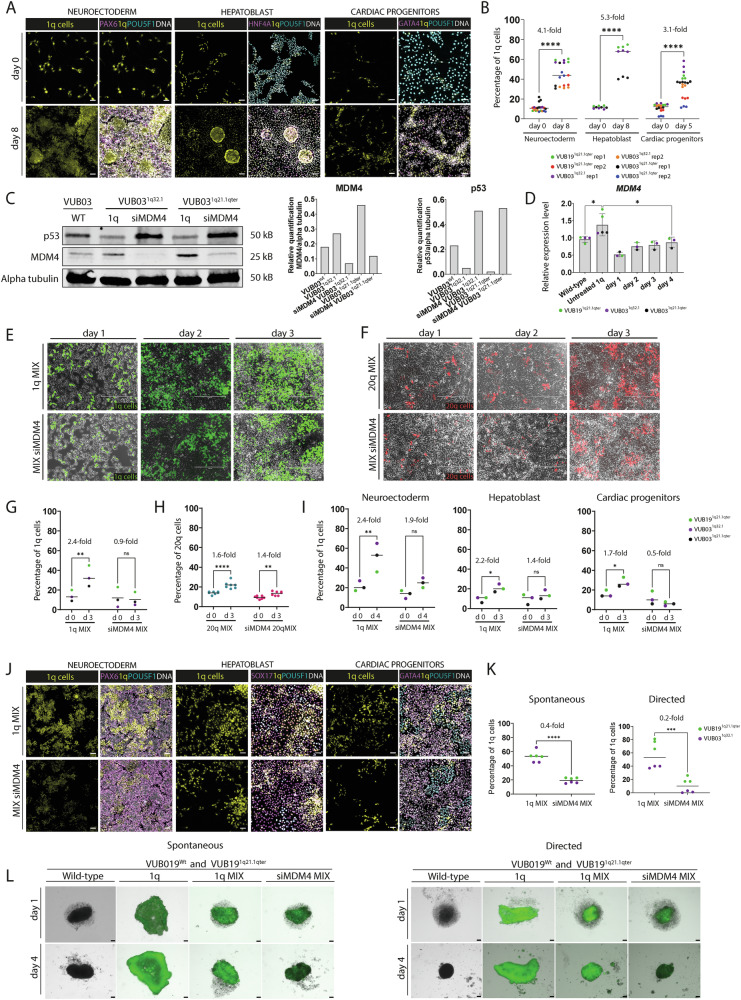
hESC^1q^ have an *MDM4*-driven competitive advantage that is retained during differentiation. **A** Representative images of immunostaining for PAX6 (magenta left panel), HNF4A (magenta, middle panel) and GATA4 (magenta, right panel) after induction of cell competition during the differentiation to neuroectoderm, hepatoblast and cardiac progenitors. Throughout all panels, 1q cells are shown in yellow, POU5F1 cells in turquoise and DNA in white. **B** Quantification of 1q-cells at the start and end of differentiation. **C** Western blot and intensity quantification of protein bands of MDM4 and p53 in hESC^wt^, hESC^1q^ and siMDM4-treated hESC^1q^. **D** Time-course of mRNA expression of *MDM4* in siMDM4-treated and untreated hESC^1q^. **E** Images of the competition assays in the undifferentiated state. hESC^1q^ (green) are mixed in at a 1:9 ratio with hESC^wt^ and traced by their fluorophore expression. In the siMDM4 condition, the hESC^1q^ have been treated with siRNA against *MDM4* prior to mixing. **F** Images of the competition assays of hESC^20q11.21^ (red) in the undifferentiated state. The experimental setup is as in panel (**E**). **G** Quantification of hESC^1q^ at the start of the cell competition (day 0) and at day 3 of in vitro culture. **H** Quantification of the numbers of hESC^20q11.21^ at the start of the cell competition (day 0) and at day 3 of in vitro culture. **I** Quantification of 1q-cells at the start of the cell competition during neuroectoderm, hepatoblast and cardiac progenitor differentiation (day 0) and at day 3 or 4, with and without siRNA against *MDM4*. **J** Representative images of immunostaining for PAX6 (magenta left panel), SOX17 (magenta, middle panel) and GATA4 (magenta, right panel) after induction of cell competition during the differentiation to neuroectoderm, hepatoblast and cardiac progenitors, with and without treatment of the hESC^1q^ with siRNA against *MDM4*. Throughout all panels, 1q cells are shown in yellow, POU5F1 cells in turquoise and DNA in white. **K** Quantification of 1q-cells in embryoid bodies after 4 days of spontaneous differentiation and directed neuroectoderm differentiation. Four EBs were pooled for dissociation and counting of fluorescently labeled 1q cells. **L** Example of embryoid bodies for cell line VUB19 on day 1 and 4 of spontaneous and directed neuroectoderm differentiation. Cells with 1q gain are labeled with green fluorescence. *p < 0.05, **p < 0.01, ****p < 0.0001, ns = non-significant.

To determine if cells with a gain of 1q retained their ability to take over the culture during differentiation, we carried out competition assays during NE, HEP and CP induction. For this, 10% of hESC^1q^ stably expressing a fluorescent protein were introduced into an unlabeled hESC^wt^ culture. Differentiation was initiated the next day and was controlled by immunostaining for PAX6, HNF4A and GATA4 (Fig. 3A). To measure culture takeover, the proportion of hESC^1q^ was determined by flow cytometry at the onset and at the end of differentiation.

We found that in all three differentiations, the cells with a gain of 1q outcompeted wildtype cells (Fig. 3B). During the NE induction, the proportion of 1q cells increased in average 33.9% ± 2.7% during the 8-day differentiation, from a mean of 10.9% at the onset to 44.9% at day 8 (*p* < 0.0001, unpaired *t*-test, *N* = 18–21). This was similar for the 8-day differentiation to HEP, where the 1q cells increased in average 49.3% ± 4.9% (mean at day 0 = 11.6%, mean at day 8 = 60.9%, *p* < 0.0001, unpaired *t*-test, *N* = 9). This increase was less pronounced during the 5-day CP differentiation, with an average 22.7% ± 3.2% increase (mean at day 0 = 10.9%, mean at day 5 = 33.5%, *p* < 0.0001, unpaired *t*-test, *N* = 18–21), which may be attributable to the shorter time span of the CP differentiation as compared to NE and HEP.

Next, we tested the role of *MDM4* in the competitive advantage of cells with a 1q gain. We first quantified MDM4 and p53 protein levels in hESC^1q^ as compared to their isogenic counterparts and tested the effect of downregulating *MDM4* by siRNA for 24 h (Fig. 3C, uncropped membranes shown in Fig. S7). Quantification of the western blot bands revealed a 1.5-fold increase in *MDM4* in VUB03^1q32.1^ and a 2.6-fold increase in VUB03^1q21.1qter^ compared to their isogenic controls. After si*MDM4* treatment, MDM4 protein decreased by 0.3-fold in both cell lines compared to hESC^1q^. Conversely, p53 levels were 0.2-fold and 0.1-fold lower in VUB03^1q32.1^ and VUB03^1q21.1qter^, respectively, compared to hESC^wt^ cells. After si*MDM4* treatment, p53 levels increased 10.2-fold and 26.5-fold compared to untreated VUB03^1q32.1^ and VUB03^1q21.1qter^ respectively.

We further controlled if the siRNA-mediated downregulation of *MDM4* in hESC^1q^ restored its mRNA levels to those of hESC^wt^ and whether this was stable over the course of 4 days post transfection (Fig. 3D). *MDM4* mRNA was 1.4-fold higher in hESC^1q^ than in hESC^wt^ (*p* = 0.0454, *N*^wt^ = 4, *N*^1q^ = 5). After 24 h of siRNA treatment, *MDM4* mRNA was reduced to 0.5-fold of the hESC^wt^ levels, and by day 4 the expression was 0.9-fold of that of wildtype cells.

We then carried out competition assays in undifferentiated and differentiating cells. For the undifferentiated cells, we downregulated *MDM4* for 24 h by siRNA in hESC^1q^ on the day prior to the start of the competition assay. The cells were imaged daily and counted at the start and end points (Fig. 3E). On average, hESC^1q^ increased from 14.0 to 33.7% in three days in the untreated condition, while no increase was observed after siRNA treatment (*N* = 3, *p*^1q^ = 0.0028, *p*^si1q^ = 0.7905, 2-way ANOVA, Fig. 3G). We carried out the same experiment on a hESC line carrying the recurrent gain of 20q11.21, which provides the cells with a Bcl-xL-mediated decreased sensitivity to apoptosis [18, 19]. Downregulation of *MDM4* did not affect the growth advantage of the hESC^20q11.21^ cells (*N* = 7, *p*^20q^ < 0.0001, *p*^si20q^ = 0.0033, 2-way ANOVA, Fig. 3F, H), showing that the suppression of the competitive advantage by modulating the *MDM4* expression is specific to cells with a gain of 1q and not due to a general decrease in cellular fitness.

For the competition assays during differentiation, the hESC^1q^ were treated for 24 h with the siRNA and mixed at a 1:9 ratio, and differentiation was initiated the next day. Differentiation was shortened to 4 days, as this was the time we found that a single siRNA transfection could reliably sustain a gene’s downregulation (Fig. 3D). In the untreated conditions of the competition assays, the fraction of cells with a gain of 1q became significantly larger in all three differentiations (Fig. 3I, J). The increase was most pronounced after NE induction, with an average increase of 30.0% (*p*^1q^ = 0.0053, 2-way ANOVA). In HEP and CP, the mean increases were 11.3% (*p*^1q^ = 0.0131, 2-way ANOVA) and 11.67% (*p*^1q^ = 0.0149, 2-way ANOVA), respectively. Conversely, in the siRNA-treated competition assays, the fraction of 1q cells remained unchanged (Fig. 3I, J). Taken together, these results show that reducing the levels of *MDM4* in the mutant cells abolishes their competitive advantage both in the undifferentiated state and during differentiation.

We next studied the competitive ability of 1q cells in a 3D differentiation using embryoid bodies (EBs, Figs. 3L, S8A, B). The EBs underwent both spontaneous and directed differentiation into neuroectoderm over a period of 4 days. For competition assays, mixtures were prepared with 10% fluorescently labeled hESC^1q^ cells or hESC^1q^ cells treated with siMDM4, as described previously. EBs were imaged daily, and the proportion of fluorescent cells was quantified at the end of the 4-day differentiation period (Fig. 3K). All EBs were characterized by RT-qPCR analysis of early differentiation markers (Fig. S8E–G). In the spontaneous differentiation conditions, at day four, EBs contained on average 52.83% 1q cells, while EBs with siMDM4-treated cells had 19.33% 1q cells (*p* < 0.0001, *N* = 6). The same experimental setup was applied for directed differentiation into neuroectoderm, where on day 4, EBs contained 56.83% 1q cells, whereas EBs with MDM4 downregulation exhibited 10.67% 1q cells (*p* = 0.0006, *N* = 6). Lastly, we carried out spontaneous differentiation by only withdrawing TGFβ and FGF2 from the cells, in monolayer cultures, which yielded similar results (Fig. S8C, D).

### Higher *MDM4* expression in hESC^1q^ results in a decreased sensitivity to DNA damage-induced apoptosis

Next, we sought to elucidate by which mechanisms higher expression of *MDM4* confers the competitive advantage to the cells. We first tested the hypothesis that the higher expression of *MDM4* by cells carrying a gain of 1q leads to a decreased p53-mediated apoptosis in response to DNA damage. For this, we induced DNA damage in hESC^1q^ and hESC^wt^ using Bleomycin and carried out a time-course measurement of apoptosis and cell death.

**Fig. 4:**
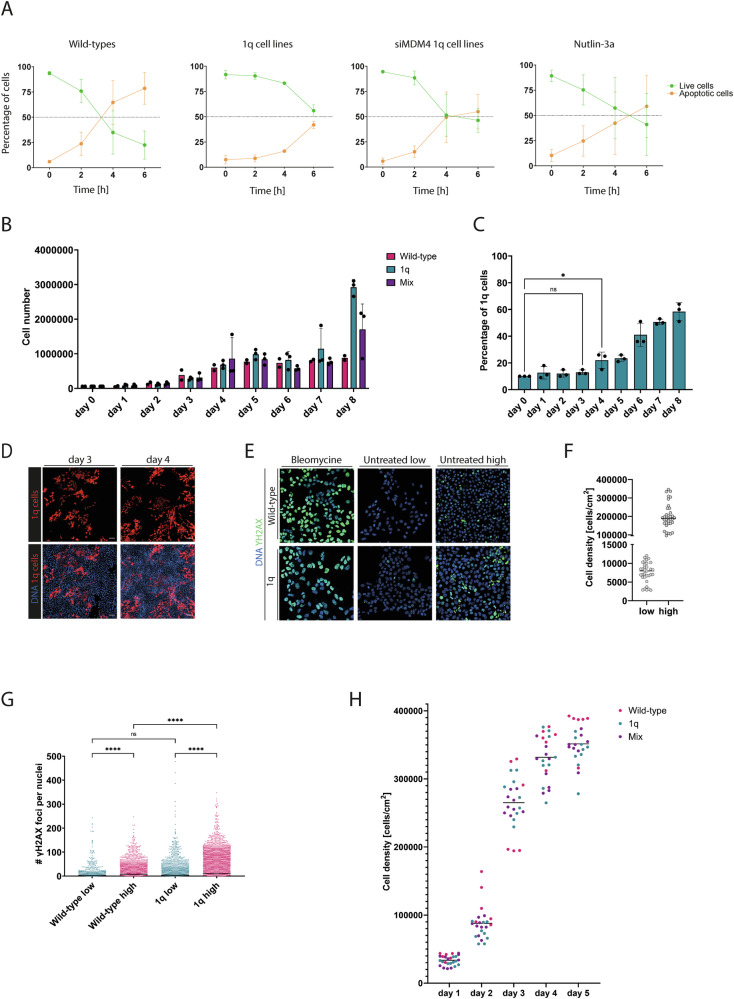
Higher MDM4 expression in hESC^1q^ results in a decreased sensitivity to DNA damage-induced apoptosis. **A** Percentages of apoptotic and live cells in hESC^wt^, hESC^1q^ and treated hESC^1q^ with siMDM4 and Nutlin-3a upon 0 h, 2 h, 4 h and 6 h of exposure to Bleomycin. **B** Evolution of the numbers of cells from day 0 to day 8, for hESC^1q^, hESC^wt^ and a 1:9 mix of hESC^1q^ and hESC^wt^. **C** Percentage of hESC^1q^ over the course of 8 days in culture, starting from a 1:9 mix of hESC^1q^ and hESC^wt^. The proportion of hESC^1q^ becomes significantly increased as from day 4. *p < 0.05. **D** Representative images illustrating the cell density at days 3 and 4 of the time-course experiment. Confluency is reached on day 4 of this experiment. 1q cells are shown in red and DNA in blue. **E** Examples of the staining for gammaH2AX (green) in hESC^1q^ and hESC^wt^, upon exposure to Bleomycin and in low- and high-density cultures. DNA is shown in blue. **F** Cell densities for the low- and high-density culture conditions shown in (**E** and **G**). The dots represent the counts made in different images of dishes of different cell lines. The line represents the mean of the dots. **G** Counts of gammaH2AX foci per nucleus in hESC^1q^ and hESC^wt^ at low and high-density culture conditions. Each dot represents a single nucleus, hESC^wt-low^ *N* = 976, hESC^wt-high^ *N* = 2879, hESC^1q-low^ *N* = 2995, hESC^1q-high^ *N* = 7158. **H** Cell densities of hESC^wt^, hESC^1q^ and mixes calculated from cell counts during a 5-day culture period.****p < 0.0001, one-way ANOVA and Šidák correction. ns = non-significant.

Figure 4A shows the percentages of live and apoptotic and dead cells for hESC^wt^ (*N* = 3), hESC^1q^ (*N* = 3), hESC^1q^ treated with siRNA against MDM4 (*N* = 3) and hESC^1q^ treated with Nutlin-3a, an inhibitor of MDM4 (*N* = 3), at the start of Bleomycin treatment and at the subsequent 2, 4 and 6-h time-points. hESC^wt^ start undergoing apoptosis 2 h after exposure to Bleomycin, followed by a rapid decrease in the numbers of live cells. In contrast, apoptotic cells start appearing in hESC^1q^ as from 4 h of exposure and reach 41.9% of apoptotic cells at 6 h, as compared to 78.6% in hESC^wt^ (unpaired *t*-test, *p* = 0.0169). Treating hESC^1q^ with siMDM4 significantly increases their sensitivity to DNA damage, with apoptosis initiating at 2 h, and reaching 49.4% at 4 h. siMDM4-treated hESC^1q^ cells do not reach same levels of dead cells at 6 h as in hESC^wt^ cells, although the differences are not statistically significant (54.9% vs 78.6%, unpaired *t*-test, *p* = 0.1530, Fig. 4A). This may be explained by an incomplete transfection of the cells, or to overall insufficiently stable downregulation of *MDM4* to the levels of hESC^wt^. Nutlin-3a treated hESC^1q^ show similar sensitivity to apoptosis as cells with downregulated *MDM4*. The apoptosis initiated at 2 h of Bleomycin treatment and reached 42.3% of apoptosis at 4 h.

This raised the question of how a decrease in sensitivity to DNA damage could provide a competitive advantage to hESC and differentiating cells. We observed during daily monitoring of the competition assays that hESC^1q^ started outcompeting their genetically balanced counterparts once the cultures became confluent. Previous work has indicated that hPSC are prone to replication stress and DNA damage [55, 56], which can be mitigated by addition of nucleosides to the medium [57] and exacerbated in higher cell density culture due to medium acidification [58, 59]. We therefore hypothesized that higher culture cell density generates the conditions for a strong competitive advantage of 1q gains, by increasing the levels of DNA damage.

We studied the cell proliferation dynamics of hESC^wt^ and hESC^1q^ over time in culture in pure form and mixed at a 1:9 ratio (*N* = 3 for each condition). Daily cell numbers count showed that the numbers of hESC^wt^ and hESC^1q^ increased similarly until day 7, suggesting that they have similar cell doubling times. As from day 8, hESC^1q^ continue proliferating even when they have reached a very high density (Fig. 4B). Daily analysis of the ratio between hESC^wt^ and hESC^1q^ showed no statistically significant changes until day 3, after which the proportion of hESC^1q^ started steadily increasing (Fig. 4C, *N* = 3). This coincided with culture dishes at day 3 still showing empty areas, whereas at day 4 the cells had reached confluence, suggesting that this is a flipping point for 1q gains to start providing a competitive advantage (Fig. 4D). To investigate the relationship between cell culture density and DNA damage, we measured DNA damage by γH2AX staining in hESC^1q^ and hESC^wt^, in low and high cell density cultures (mean of 7547 cells/cm^2^ and 200,050 cm/cm^2^ respectively, representative images are shown in Fig. 4E and cell counts shown in Fig. 4F), and found that cells grown at low density had significantly lower numbers of γH2AX foci than when grown in high density (Fig. 4G, wt_low_: 11.38, wt_high_: 17.95, 1q_low_: 13.71, 1q_high_: 26.22, *p* < 0.0001, one-way ANOVA). While there were no differences in DNA damage between hESC^1q^ and hESC^wt^ grown at low density (Fig. 4G, 1.2-fold change, *p* = 0.2314, one-way ANOVA), at high density, hESC^1q^ showed higher γH2AX foci counts than hESC^wt^ (Fig. 4G, 1.5-fold change, *p* < 0.0001, one-way ANOVA). Lastly, we counted the numbers of cells in the cell cultures until day 5 and calculated the densities (Fig. 4H). The mean densities in days 1 and 2 are in the range of the ‘low density’ group in the DNA damage staining, with a mean of 33,711 cells/cm^2^ at day 1 and 87,854 cells/cm^2^ at day 2. From day 3, the densities are in the range and above of the ‘high density’ group, with 265,224 cells/cm^2^ at day 3, 331,680 cells/cm^2^ at day 4 and 351,475 cells/cm^2^ at day 5. This indicates that at this point, the cells start undergoing DNA damage more frequently, reaching the condition when a decreased p53-mediated induction of apoptosis starts providing a selective advantage, so cells with a gain of 1q will start outcompeting their genetically balanced counterparts.

## Discussion

Despite that, the appearance of abnormal karyotypes in hESC was first noticed nearly 20 years ago [60] we still have limited insight into their mechanisms of origin, their driver genes and their functional consequences. In this study, we find that hESC^1q^ have an abnormal response to the differentiation cues to neuroectoderm, with a part of the population mis-specifying to non-CNS cells. Also, while entering the mesendodermal lineage equally efficiently as hESC^wt^, hESC^1q^ generate hepatoblasts and cardiac progenitors that have gene-expression profiles suggestive of poor cell maturation. It is interesting to note that hESC with a gain of 20q11.21 [24, 61], an isochromosome 20 [62] and with a loss of 18q [63] all display an abnormal response to the neuroectoderm differentiation signaling cues. This common response suggests a particular vulnerability of the neuroectodermal lineage to genetic abnormalities, with potentially common signaling pathways disrupting the differentiation process. In this study, we have identified the alternative cell fate as non-CNS ectoderm, the cells expressing markers of cranial placode and non-neural ectoderm. These cell types are not commonly obtained in the standard NE differentiation by dual-SMAD inhibition, but it has been shown that the addition of different concentrations of BMP4 and modulation the WNT pathway will drive the cells towards these fates [39]. Further, it has been shown that DNA damage-induced stabilization of p53 in hPSC results in activation of TGF-beta signaling and a downregulation of BMP4 pathway genes [64], suggesting that deregulation of these pathways specifically in hESC^1q^ during the differentiation process may be at the basis of this alternate fate acquisition.

The differentiation of mesendoderm cell types yielded similar proportions of correctly specified cells, but gene-expression analysis revealed that the hepatoblasts and cardiac progenitors obtained from hESC^1q^ had profiles suggestive of a more immature cell types. Whether this reflects delayed or impaired maturation and whether this extends to other mesendoderm derivates remains to be elucidated, but it further supports the notion that genetic abnormalities do affect the quality of the cells obtained after differentiation, which can negatively impact both the clinical translation of hPSC as well as the reproducibility of results when using these cells in research [16]. Further, here we also show that the genetically variant cells retain their ability to take over a culture during differentiation. Considering that, 10 to 20% of cells in an hPSC culture carry a variety of copy number variations [58, 65, 66], and 10% of hESC lines carry mosaic large structural variants [13], it is possible that the differentiation of a seemingly genetically normal line yields a mixed population of euploid and aneuploid cells with varying degrees of correct cell specification. This lays a further layer of complexity into controlling the outcomes of differentiation, as current methods for genetic screening will easily miss low-grade mosaicism.

In this work, we identify *MDM4* as the driver gene behind the competitive advantage of cells with a gain of 1q, finding that this advantage stems from reduced sensitivity to DNA damage-induced apoptosis due to *MDM4*’s suppression of p53 activity. We found that culture conditions were instrumental in creating the environment in which mutant cells outcompete their wild-type counterparts, with cell density in culture playing a key role. In line with this, Stavish et al. recently reported that feeder-free stem cell culture such as E8/vitronectin can increase susceptibility to genomic damage, as compared to feeder-layer systems. This, in turn, favors the culture takeover by cells with a gain of 1q [67]. Together, these results open the possibility of tailoring culture conditions to suppress mutant cell takeover, such as by controlling cell density, regulating the pH of the culture medium to reduce DNA damage [58, 59] or adding factors such as nucleosides [57].

Finally, *MDM4* has been shown to also be the driver of the addiction of cancers to chromosome 1q gains to support their malignant growth [68]. The similarities between the genetic abnormalities found in cancers and hPSC have been already noted in the past [69–71]. Gains of 20q11.21, one of the most common acquired abnormalities in hPSC [11], appear in over 80% of pancreatic and colorectal carcinomas [72, 73] and gains of 1q appears in over half of the cases of hepatocellular carcinoma [74] and have been recently identified as the first copy number alterations occurring in breast cancer and melanoma evolution [68]. These parallels, along with the frequent gain of 1q in culture, the fact that the variant cells can take over also during differentiation and the impact on the final cell product, highlight the need to consider genetic integrity in both clinical and research settings to ensure reliable and reproducible results [75, 76].

## Materials and methods

### hESCs lines, cell culture and banking and transgenic modification

All the hESC lines were derived from human embryos and characterized as described in [77, 78]. They are registered in the EU hPSC registry (https://hpscreg.eu/). VUB03 and VUB19 remained genetically balanced up to passage 20 and 24, respectively. All stocks of hESC lines are preserved in liquid nitrogen in 90% Knock-out serum (Thermo Fisher) and 10% sterile DMSO. hESC were cultured in dishes coated with human recombinant Laminin-521 (Biolamina). Laminin-521 was diluted in phosphate-buffered saline (PBS) (Thermo Fisher) with calcium and magnesium. Coated dishes were stored overnight at 4 °C before the use. NutriStem™ (Biological Industries) medium with 10 mM penicillin/streptomycin was changed daily. hESC cultures were kept in the incubator at 37 °C and 5% CO_2_. For passaging, cells were washed with PBS and incubated with recombinant enzymes TrypLE (Thermo Fisher) for 10 min at 37 °C for single-cell dissociation. TrypLE was deactivated with NutriStem™ medium and cells were centrifuged at 1000 rpm for 5 min. Pellet was resuspended in 1 ml of medium and counted with image-based cytometer Tali™ (Invitrogen) if needed or transferred in a new dish in ratio between 1:20 and 1:50.

Labeled hESC^1q^ cells were generated by infecting hESC^1q^ with lentiviral particles expressing fluorescent protein. HEK293T cells were transfected with pMDG (VSV.G encoding plasmid), pCMVΔR8.9 (gag-­‐pol encoding plasmid) as packing vectors and three different vectors each with their own fluorescent protein (LeGO-­‐EF1a-­‐V2-­‐Puro (Venus fluorescent protein)), LeGO-­‐EF1a-­‐C2-­‐Puro (mCherry) and pCDH-­‐EF1-­‐MCS-­‐pA-­‐PGK-­‐copGFP-­‐T2A-­‐Puro (Green fluorescent protein). Vectors with mCherry and Venus fluorescent protein were kindly provided by Kristoffer Riecken [79]. Vector with GFP was a gift from VUB Laboratory for molecular and cellular therapy. Vector was purchased from Addgene (https://www.addgene.org/17448/) and cloned CMV promoter to EF1a. Lentiviruses were produced by transfection of HEK293T cells with VSV.G and gag-pol plasmids together with the plasmid of interest and PEI (2 µg per µg of DNA, Polysciences Inc) in Opti-MEM medium (Thermo Fisher). After 4 h of transfection, the transfection cocktail was replaced by complete medium. Supernatant containing lentiviral particles was collected after 48 and 72 h and stored at −80 °C. A day before hPSC were seeded at the density 5000 cells/cm^2^. hESC were transduced with 1:1 mix of Nutristem and complete medium containing lentivirus together with 1:1000 protamine sulfate (LEO Pharma; 10 mg/ml). Cells were incubated with transduction cocktail for 4 h. Next, cells were washed 5 times with PBS and refreshed with Nutristem medium. Selection of successfully transduced cells was done with FACS.

Working cell banks comprising labeled 1q cell lines and their isogenic counterparts were established, which were additionally controlled for cell identity and karyotype. We utilized the working cell bank for subsequent experiments and the cells were not kept in culture for more than 5 passages after thawing to prevent them from genetic drift.

### Genome characterization

Genomic DNA was extracted with DNeasy Blood & Tissue Kit (Qiagen) following the producer’s instructions. Concentration was assessed with NanoDrop 1000 (Thermo Fisher). DNA was stored at 4 °C. The genetic content of the hESCs was assessed through shallow whole-genome sequencing by the BRIGHTcore of UZ Brussels, Belgium, as previously described [80]. Copy-number assays were used to control for gains of 1q, 12p and 20q11.21 at regular intervals. Real-time polymerase chain reaction was performed on ViiA™ 7 system (Applied Biosystems). Total volume of qPCR reaction was 20 µl and it contained 10 µl of 2x qPCR Master Mix Low ROX (Eurogentec), 1 µl of 20x TaqMan Copy Number Assay (Life Technologies), 1 µl of nuclease-free water and 40 ng of DNA. TaqMan™ Copy Number Reference Assay RNase P (Applied Biosystems, 4403328) was added instead of nuclease-free water. Genomic DNA from leukocytes of healthy donor was used as a control. Non template control was tested in each experiment. Cycling parameters are 2 min at 50 °C, 40 cycles with 10 min at 95 °C and 15 s at 95 °C and 1 min at 60 °C. Each sample was tested in triplicates. Copy number assays used were KIF14 (Thermo Fisher, Hs00637799_cn), NANOG (Thermo Fisher, Hs03820140_cn) and ID1 (Thermo Fisher, Hs01892845_cn). Experiments were run as either comparative Ct (cycle threshold) or relative standard curve analysis. Data analysis was performed by ViiATM 7 v2.0 software or Copy Caller v2.1 (Applied Biosystems).

### Neuroectoderm differentiation

The protocol for neuroectoderm induction was adapted from Douvaras et al. [81]. We seeded 22,500 cells/cm^2^ on dishes coated with Laminin-521 and 1:100 RevitaCell (Thermo Fisher). After 24 h, differentiation was induced with freshly prepared neural induction medium containing DMEM/F12 (Thermo Fisher), 1x NEAA (TFS), 1x GlutaMAX (TFS), 1×2-mercaptoethanol (TFS), 25 µg/ml insulin (Sigma-Aldrich) and 1x penicillin/streptomycin (TFS). The medium was supplemented with 10 μM SB431542 (Tocris), 250 nM LDN193189 (STEMCELL Technologies) and 100 nM RA (Sigma-Aldrich). The medium was refreshed every day for 8 days.

### Cardiac progenitor differentiation

The protocol for cardiac progenitor differentiation was modified from Lin et al. [82]. hESC were seeded at the density of 22,500 cells/cm^2^ on Laminin-521 coated plates. RevitaCell was added to the cell suspension in 1:100 dilution before the seeding. When cells reached 90% confluence, differentiation was initiated with cardiomyocyte differentiation basal medium (CDBM) supplemented with 5 µM CHIR99021 (Tocris). After 24 h treatment with CHIR99021, medium was changed with fresh CDBM with addition of 0.6 U/ml heparin (Sigma-Aldrich). For the next 3 days, CDBM with 0.6 U/ml heparin and 3 mM IWP2 (Tocris) was refreshed daily. CDBM medium was composed of DMEM/F12 (Thermo Fisher), 64 mg/l L-ascorbic acid (Sigma-Aldrich), 13.6 µg/L sodium selenium (Sigma-Aldrich), 10 µg/ml transferrin (Sigma-Aldrich) and 1x chemically defined lipid concentrate (Thermo Fisher).

### Hepatoblast differentiation

The hepatoblast differentiation protocol was based on protocol from Boon et al. [83]. hESC were seeded with 1:100 RevitaCell (Thermo Fisher) at the density of 35,000 cells/cm^2^ on Laminin-521 coated plates. Next day Nutristem medium was refreshed. Differentiation was initiated the next day with liver differentiation medium (LDM) supplemented with 50 ng/ml Activin A (STEMCELL Technologies), 50 ng/ml WNT3A (PreproTech) and 6 µl/ml DMSO (Sigma-Aldrich). After 48 h, medium was refreshed without WNT3A factor. On days 4 and 6 LDM medium with added 50 ng/ml BMP4 (STEMCELL Technologies) and 6 µl/ml DMSO was used. LDM medium was composed of MCDB 201 medium with pH 7.2 (Sigma-Aldrich), DMEM High Glucose medium (Westburg Life Sciences), L-Ascorbic Acid (Sigma-Aldrich), Insuli-Transferrin-Selenium (ITS-G, Thermo Fisher Scientific), Linoleic Acid-Albumin (LA-BSA, Sigma-Aldrich), 2-Mercaptoethanol (Thermo Fisher Scientific) and Dexamethasone (Sigma-Aldrich).

### Total RNA isolation, cDNA synthesis and quantitative real-time PCR

RNA was extracted from cell pellets with RNeasy Mini Kit (Qiagen) following the producer’s protocol. Concentration of obtained RNA was measured with NanoDrop 1000 (Thermo Fisher). RNA was stored at −80 °C. Reverse transcription of RNA to cDNA was performed with First-Strand cDNA Synthesis Kit (GE Healthcare) following the producer’s instructions. cDNA was stored at −20 °C.

Real-time qPCR was performed on ViiA™ 7 system (Applied Biosystems). Total volume of qPCR reaction was 20 µl and it contained 10 µl of 2x qPCR Master Mix Low ROX (Eurogentec), 1 µl of 20x TaqMan Gene Expression Assay (Life Technologies), 1 µl of nuclease-free water and 40 ng of cDNA. Each sample was tested in triplicates, GUSB was used as a housekeeping gene. Genes and assays are listed in Table S4. We used the standard cycling parameters of the Viia7 instrument. Experiments were run as comparative Ct (cycle threshold) analysis. Data analysis was performed by ViiATM 7 v2.0 software.

### Immunofluorescent staining

Cells were washed 3 times with PBS and incubated for 15 min with 3.6% paraformaldehyde at room temperature for fixation. Cells were washed again 3 times with PBS. Blocking was performed with 1–2 h incubation with 10% fetal bovine serum (FBS). Primary antibodies were diluted in specific ratio in 10% FBS and incubated with cells for 1 h in the dark at room temperature. After washing 3 times with PBS, secondary antibodies were diluted in FBS together with 1:2000 dilution of Hoechst 33342 (Life Technologies) and incubated for 2 h at room temperature. Cells were again washed three times with PBS and stored at 4 °C until analyzed with confocal microscope Zeiss. List of antibodies is shown in Table S4.

### Flow cytometry and Annexin V staining

The hESC were harvested by incubating them 10 min at 37 °C with 1 ml of TrypLE (Thermo Fisher). The cell suspension was gently pipetted up and down with 2 ml of medium and strained through 20 µm cell strainer (pluriStrainer) to eliminate any cell clumps. Single-cell suspension was centrifuged at 1000 rpm for 5 min. Medium was aspirated, cells were resuspended in 1 ml of PBS and spun down at 1000 rpm for 5 min. If needed, the cells were incubated with Alexa Fluor® 647 Annexin V (BioLegend) based on the manufacturer’s instructions. Next, the cells were washed with 1 ml of PBS and centrifuged. Incubation with Live/dead stain (Thermo Fisher) was performed following the producer’s protocol. Cells were washed again and fixed with 3.6% paraformaldehyde at room temperature for 10 min. After centrifugation (1000 rpm for 5 min) the paraformaldehyde was aspirated, the cells were washed with 1 ml of PBS and spun down. The fixed cells were resuspended in PBS and stored at 4 °C until the analysis with Flow cytometer Aria III (BD).

### Nutlin-3a experiment

hESC^1q^ were treated with 2.5 µM Nutlin-3a (Tocris) for 24 h. After removing Nutlin-3a, DNA damage and apoptosis were induced by treating the cells with 100 µg/ml of Bleomycin (Thermo Fisher). Cells were collected every 2 h, stained with Annexin V and PI (Thermo Fisher), and the percentage of positive cells was quantified.

### RNA sequencing

RNA-seq library preparation was performed using QuantSeq 3′ mRNA-Seq Library Prep Kits (Lexogen) following Illumina protocols. Sequencing was performed on a high-throughput Illumina NextSeq 500 flow cellThe FastQC algorithm [84] was used to perform quality control on the raw sequence reads prior to the downstream analysis. The raw reads were aligned to the new version of the human Ensembl reference genome (GRCh38.p13) with Ensembl (GRCh38.83gtf) annotation using STAR version 2.5.3 in 2-pass mode [85]. The aligned reads were then quantified, and transcript abundances were estimated using RNA-seq by expectation maximization (RSEM, version 1.3.3) [86].

The count matrices were imported into R software (version 3.3.2) for further processing. The edgeR [87] package was utilized to identify differentially expressed genes (DEGs) between groups. Transcripts with a count per million (cpm) greater than 1 in at least two samples were considered for the downstream analysis. Genes with a log2-fold change greater than 1 or less than −1 and a false discovery rate (FDR)-adjusted *p*-value less than 0.05 were considered significantly differentially expressed. Volcano plots of DEGs were generated using the ggplot2 [88] package in R.

Principal component analysis (PCA) and heatmap clustering were performed using normalized counts and R packages. The heatmap was generated using the heatmap.2 function. PCA was performed using the prcomp function and plotted using ggplot2. Gene set enrichment analysis (GSEA), gene ontology enrichment analysis and reactome pathway gene set enrichment analysis was applied to detect the enrichment of pathways using the fgseaMultilevel, gseGO and enrichPathway function in R. Values are ranked by sign(logFC)*(-log10(FDR)). |NES| > 0 and adjusted *p*-value < 0.05 were considered the thresholds for significance for the gene sets.

### Downregulation of MDM4 gene with siRNA

SMARTpool siRNA for MDM4 was purchased from Horizon Discovery. 1q hESC were seeded at the density 40,000 cells/cm^2^. After 24 h cells were transfected with 50 nM SMARTpool siRNA in Nutristem medium without antibiotics together with RNAiMAX (Thermo Fisher Scientific), according to manufacturer’s instructions. Transfection cocktail was added to the cells 24 h prior to seeding for differentiation.

### Formation of embryoid bodies

Embryoid bodies (EBs) were generated as previously described in [89, 90]. Briefly, hESCs were passaged two days prior to EB formation, achieving a final confluence of 60–80%. For conditions involving MDM4 downregulation, hESC^1q^ were treated with siRNA against MDM4 24 h before EB formation. The hESCs were harvested using TrypLE to obtain single-cell suspension. Next, 18,000 cells were plated into each well of an ultra-low attachment 96-well plate (Corning) in medium containing ROCK inhibitor Y-27632 (Tebubio) and low concentrations of bFGF (Peprotech) to promote spontaneous differentiation. For guided neuroectoderm differentiation, the medium was supplemented with LDN1931189 and SB-43152 for dual SMAD inhibition. Mixes consisted of 10% hESC^1q^ and 90% hESC^wt^. EBs were maintained in culture for four days before being collected for analysis.

### Western blot

For protein isolation, cells were washed three times with PBS and harvested using TrypLE for 10 min at 37 °C. The TrypLE was inactivated by adding an equal volume of medium, and the cells were centrifuged at 1000 rpm for 5 min. The cell pellet was resuspended in RIPA buffer (Sigma-Aldrich) and incubated for 20 min at 4 °C with occasional vortexing to lyse the cells. The mixture was then centrifuged at 10,000 rpm for 30 min at 4 °C. The supernatant was collected, and protein concentration was measured using iMark™ Microplate Absorbance Reader (Bio-Rad). For Western Blot, 20 µg of protein was diluted 1:4 with Laemmli buffer (Bio-Rad) supplemented with 10% β-mercaptoethanol (Sigma-Aldrich). The samples were incubated at 95 °C for 5 min, cooled on ice, and spun down before loading into precast TGX gels. The gels were run in 1X TGS buffer in a Criterion Vertical Electrophoresis Cell at 200 V for 45 min. Precision Plus Protein ladder was used to determine protein size. Protein was transferred to a nitrocellulose membrane using the Trans-Blot Turbo system (Bio-Rad). The membrane was washed with PBS-T (PBS with 0.1% Tween-20), blocked in 5% non-fat milk for 30 min, washed again, and incubated with the primary antibody in PBST overnight at 4 °C on a shaker. The next day, the membrane was washed three times for 5 min in PBST and then incubated with the secondary antibodies for 1 h at room temperature on a shaker, followed by three more 5-min washes in PBST. The samples were imaged using an Odyssey FC Imaging System (LI-COR), and band intensities were quantified using ImageJ. A list of antibodies used can be found in the supplemental information.

### Immunostaining quantification

Immunostaining quantification was performed following the protocol detailed in Measuring Early Germ-Layer Specification Bias in Human Pluripotent Stem Cells. Briefly, confocal microscopy images of immunostained cells were captured and imported into Zen Blue software (Zeiss) for analysis. A class was assigned to the fluorescent channel labeling the nuclei, while differentiation or pluripotency markers were categorized into respective classes and subclasses. Filters were then applied to facilitate accurate segmentation. Image segmentation parameters were customized for each cell line and differentiation marker to optimize analysis. Masks were generated to closely approximate the boundaries of positive nuclei. This step ensures that only relevant fluorescent signals are considered in downstream analyses. Once the masks were defined, either the signal intensity of the fluorescent markers or the number of positively stained nuclei was quantified. These metrics were then used to assess the level of marker expression across the samples.

### Statistics

All differentiation experiments were carried out in at least triplicate (*n* ≥ 3). All data are presented as the mean ± standard error of the mean (SEM) or standard deviation (SD). Statistical evaluation of differences between 2 groups was performed using unpaired two-tailed *t*-tests, one-way or two-way ANOVA in GraphPad Prism9 software, with *p* < 0.05 determined to indicate significance.

## Supplementary information

Supplementary data

Table S1

Table S2

Table S3

Table S4

Table S5

Table S6

Uncropped Westernblot Figure 3C

Uncropped Westernblot Figure 3C

## Data Availability

Raw sequencing data of human samples is considered personal data by the General Data Protection Regulation of the European Union (Regulation (EU) 2016/679) because SNPs can be extracted from the reads and cannot be publicly shared. The data can be obtained from the corresponding author upon reasonable request and after signing a Data Use Agreement. The RNA sequencing counts per million tables are provided in the supplementary material. Source data can be retrieved from osf.io/qzyp4. Further information and requests for resources should be directed to the corresponding author, CS (claudia.spits@vub.be).

## Materials availability

All VUB stem cell lines in this study, including the genetically abnormal sublines and genetically modified lines, are available upon request and after signing a material transfer agreement.
