# Supplementary material for: Gain of 1q confers an MDM4-driven growth advantage to undifferentiated and differentiating hESC while altering their differentiation capacity
Source: Cell Death Dis. 2024 Nov 21;15(11):852. doi: 10.1038/s41419-024-07236-x (PMC11582570; doi:10.1038/s41419-024-07236-x)
Supplement: Supplementary file 1 — Supplementary data [file 41419_2024_7236_MOESM1_ESM.docx]

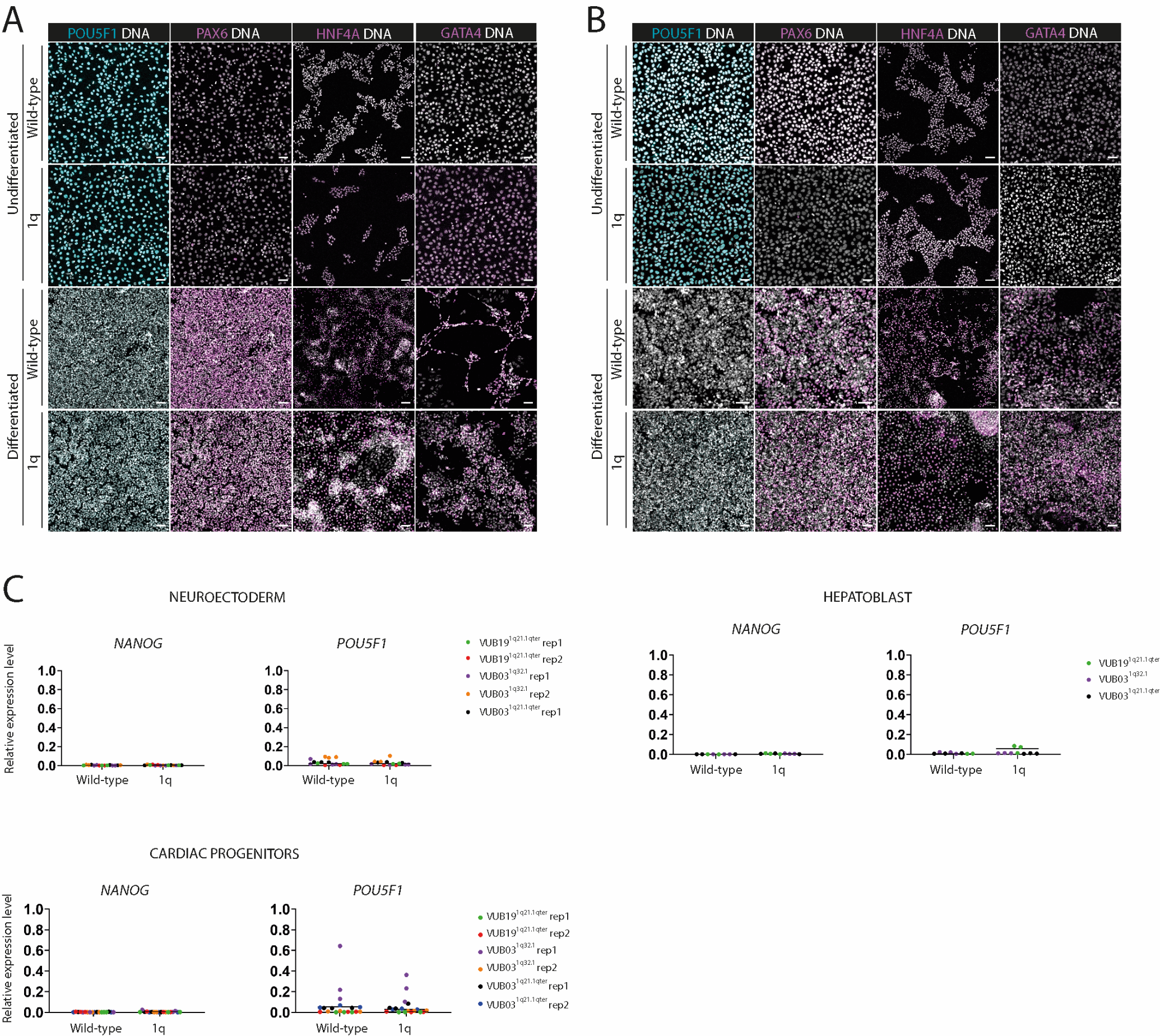


**Figure S1. Differentiation of hESC to neuroectoderm, hepatoblast and cardiac progenitors results in downregulation of pluripotency markers and upregulation of differentiation markers in wild-type and 1q cells.**

**A** Immunostaining of DNA (white), pluripotency marker POU5F1 (turquoise, 1^st^ panel) and neuroectoderm marker PAX6 (magenta, 2^nd^ panel), hepatoblast marker HNF4A (magenta, 3^rd^ panel) and cardio progenitor marker GATA4 (magenta, 4^th^ panel) of cell lines VUB03^wt^, VUB03^1q32.1^. Markers are shown in wild-type cell line and their 1q counterparts before and after the differentiation.

**B** Immunostaining in cell lines VUB03^wt^ and VUB03^1q21.1qter^.

**C** Expression of pluripotency markers *NANOG* and *POU5F1* determined with qPCR in two wild-type and three 1q cell lines. Expression is shown relative to wild-type hESC. Each independent experiment replicate (rep) is coded with a unique color.


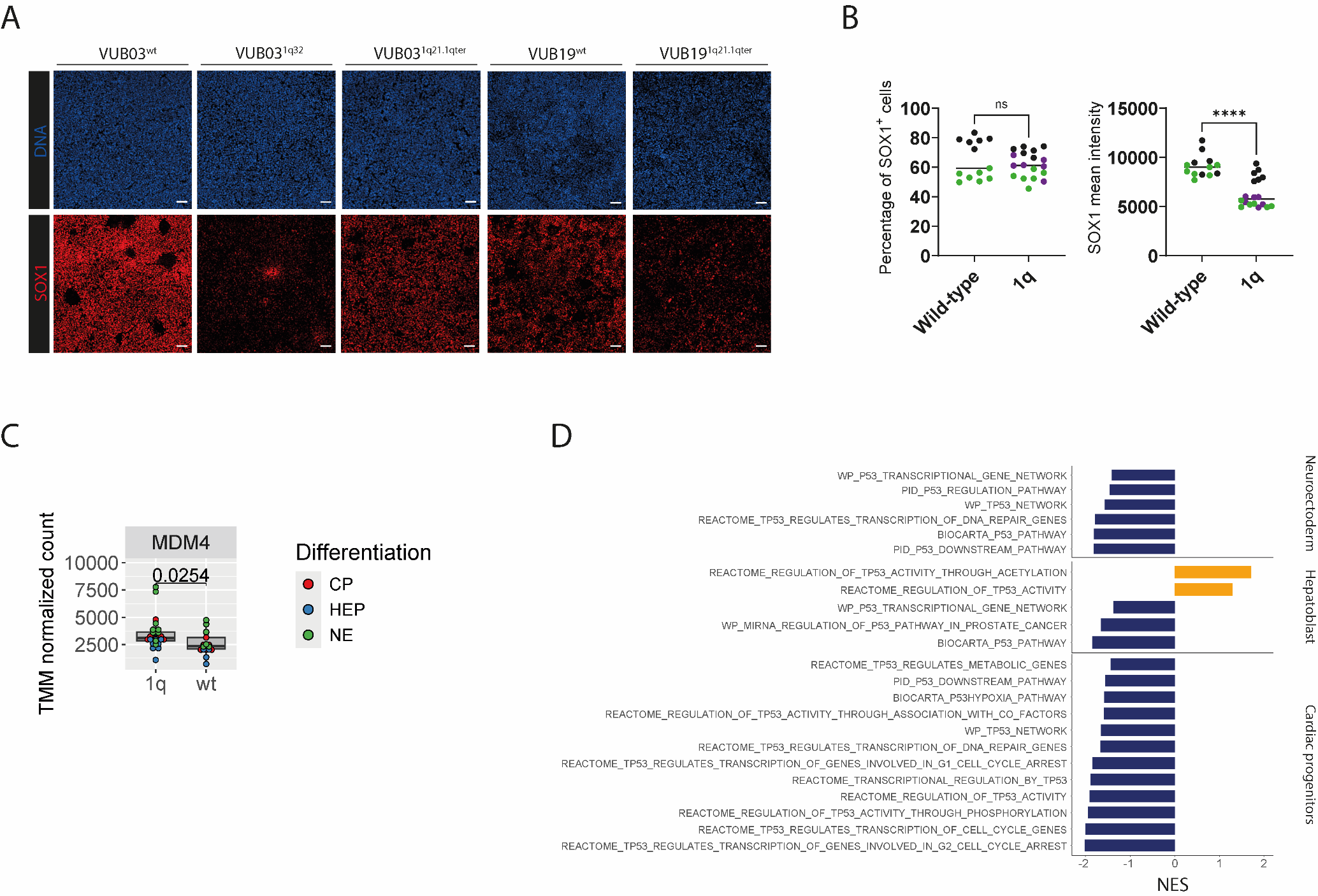


**Figure S2. Immunostaining and quantification of SOX1-positive cells, TMM count of MDM4 and GSEA results for 53-related pathways.**

**A** Immunostaining of SOX1 (red) protein after 8 days of neuroectoderm differentiation. DNA staining is shown in blue.

**B** Quantification of SOX1-positive cells and mean intensity of SOX1 expression.

**C** TMM normalized count of *MDM4* in wild-type and 1q cell lines during three-lineage differentiation.

**D** Negative normalized enrichment score in p53-related pathways in all differentiation lineages.


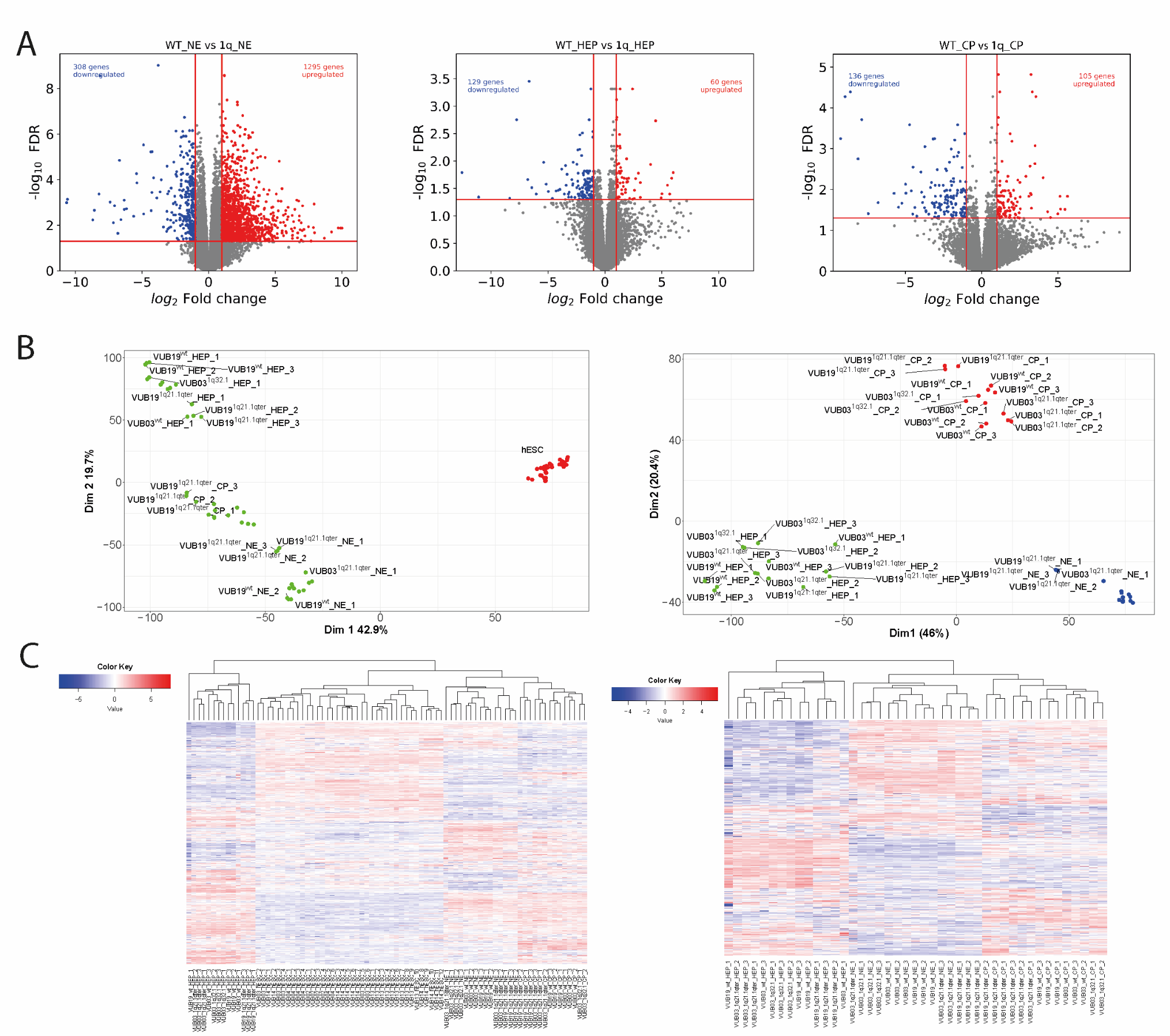


**Figure S3. Volcano plots, PCA and unsupervised heatmaps of the mRNA sequencing of NE, CP and HEP from hESC^wt^ and hESC^1q^**

**A** Volcano plots represent differentially expressed genes after neuroectoderm, hepatoblast and cardiac progenitor differentiation. Results are shown as 1q cells relative to wild-type differentiated cells. Significant results are considered at |log_2_ fold change|>1 and FDR< 0.05. In neuroectoderm differentiation 308 genes are downregulated and 1295 upregulated, in hepatoblast 129 downregulated and 60 upregulated genes and in cardiac progenitor cells 136 downregulated and 105 upregulated genes.

**B** PCA results of wild-type and 1q cell lines differentiated to 3 germ layers in relation to hPSC (left) and differentiated wild-type cells in relation to differentiated 1 q cell lines. Cell lines from the same lineage are clustering together.

**C** Unsupervised heatmap of all differentially expressed genes in differentiated and hESC lines (left) and only differentiated cell lines (right).

**
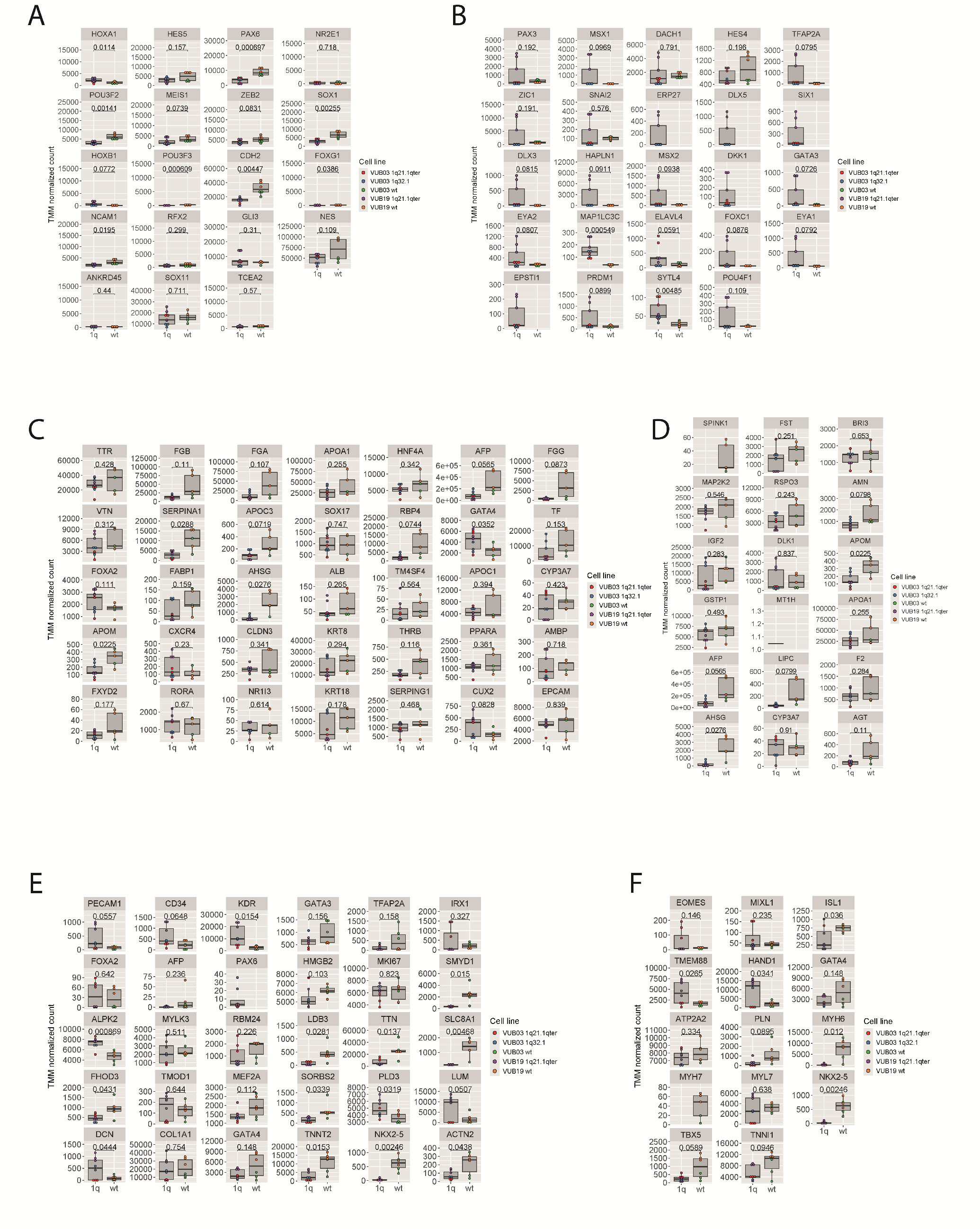
**

**Figure S4. Box plot of TMM normalized counts per gene in individual cell lines of neuroectoderm differentiation (A), non-neural ectoderm and placodes (B), hepatoblast (C) hepatoblast differentiation stage, cardiac differentiation (D) and cardiomyocyte differentiation timecourse (E). Genes correspond to lollipop diagrams in Figure 2. P-values are result of unpaired t test.**


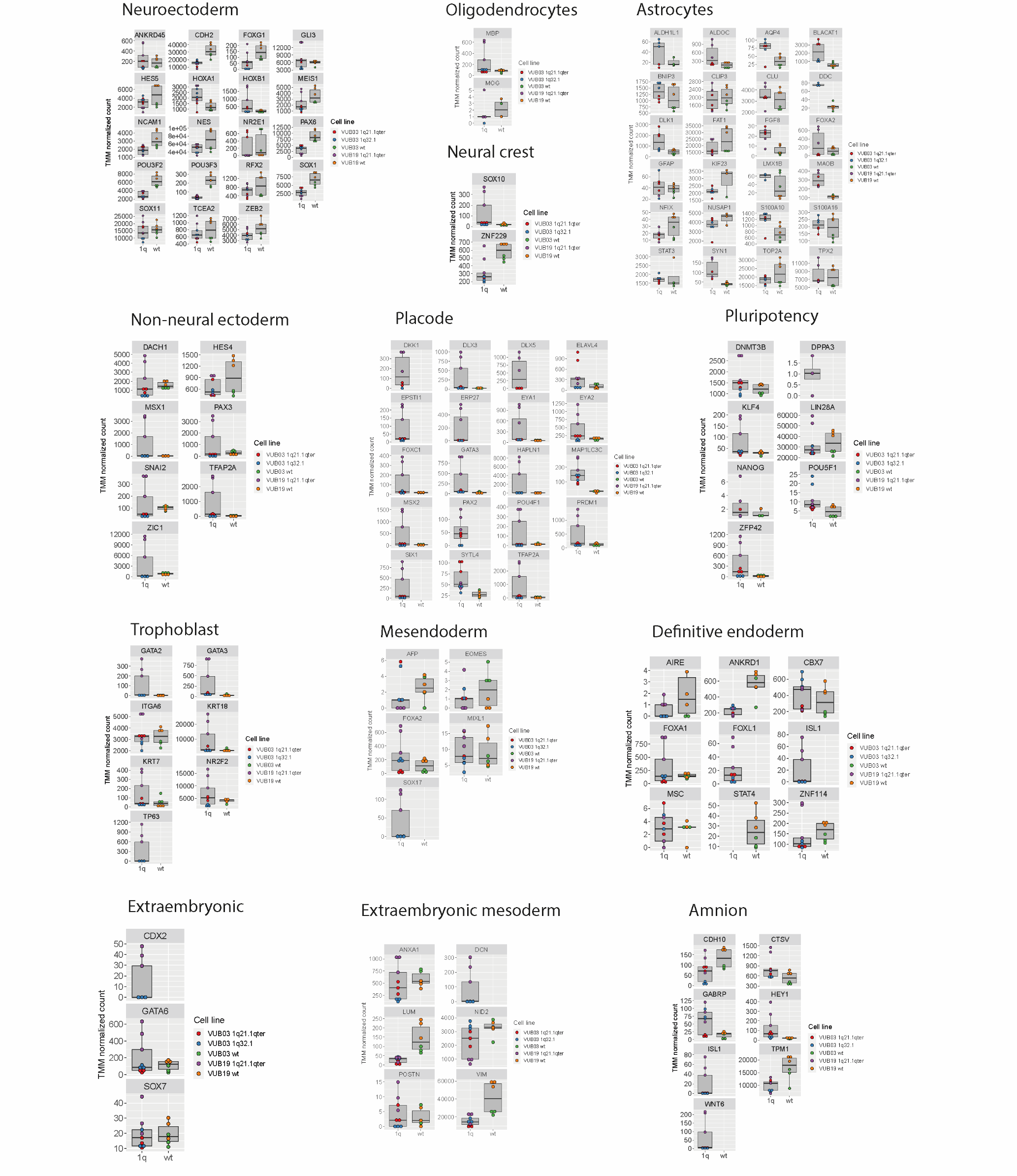


**Figure S5. Box plot of TMM normalized counts per gene in individual cell lines of neuroectoderm differentiated wild-types and 1q cell lines. Expression of different cell type markers is represented.**


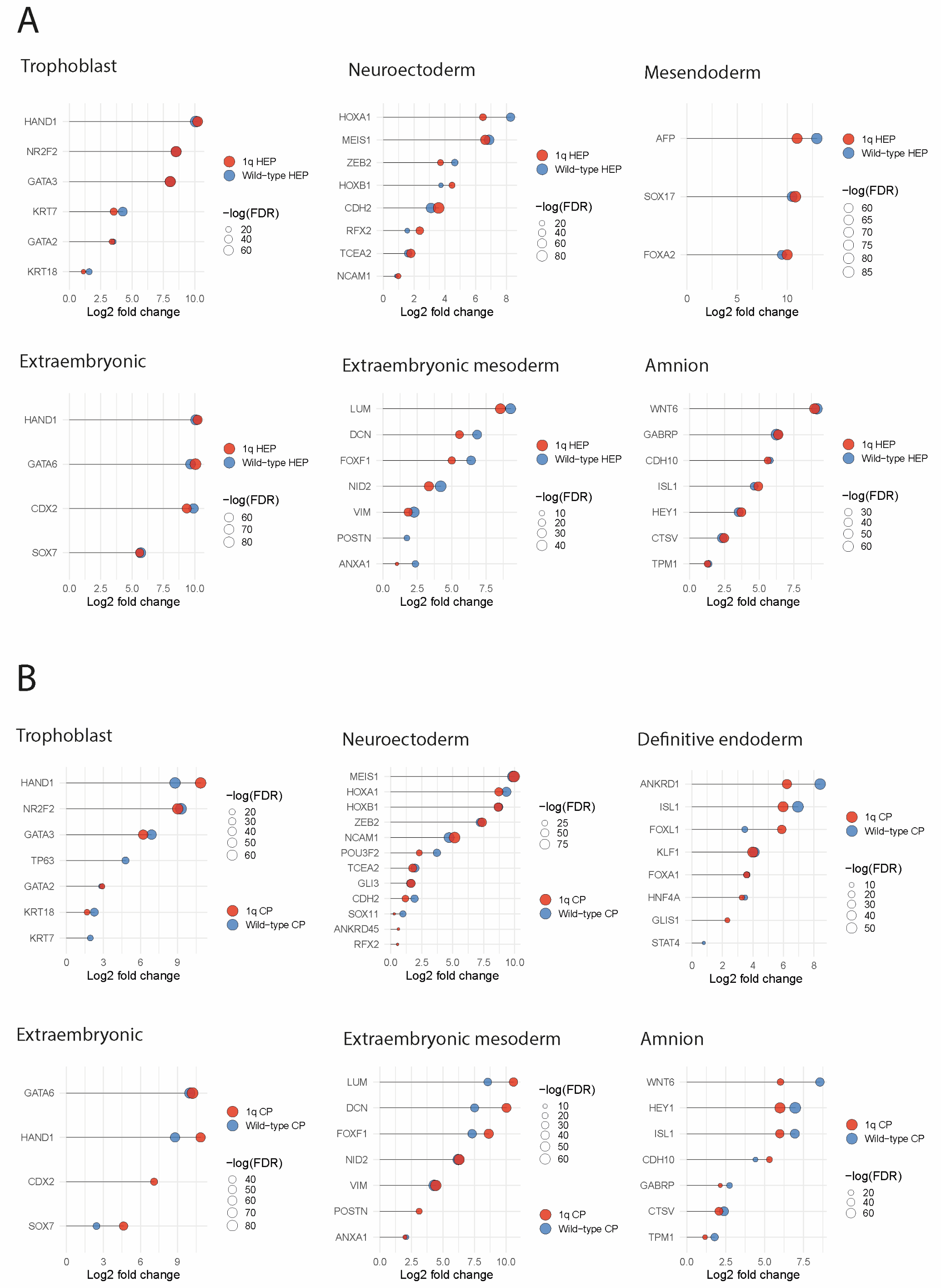


**Figure S6. Studying mis-specification in hepatoblast (A) and cardiac progenitor (B) wild-type and 1q cell lines. Lollipop plots showing log_2_ fold change expression of different cell type markers.**

**
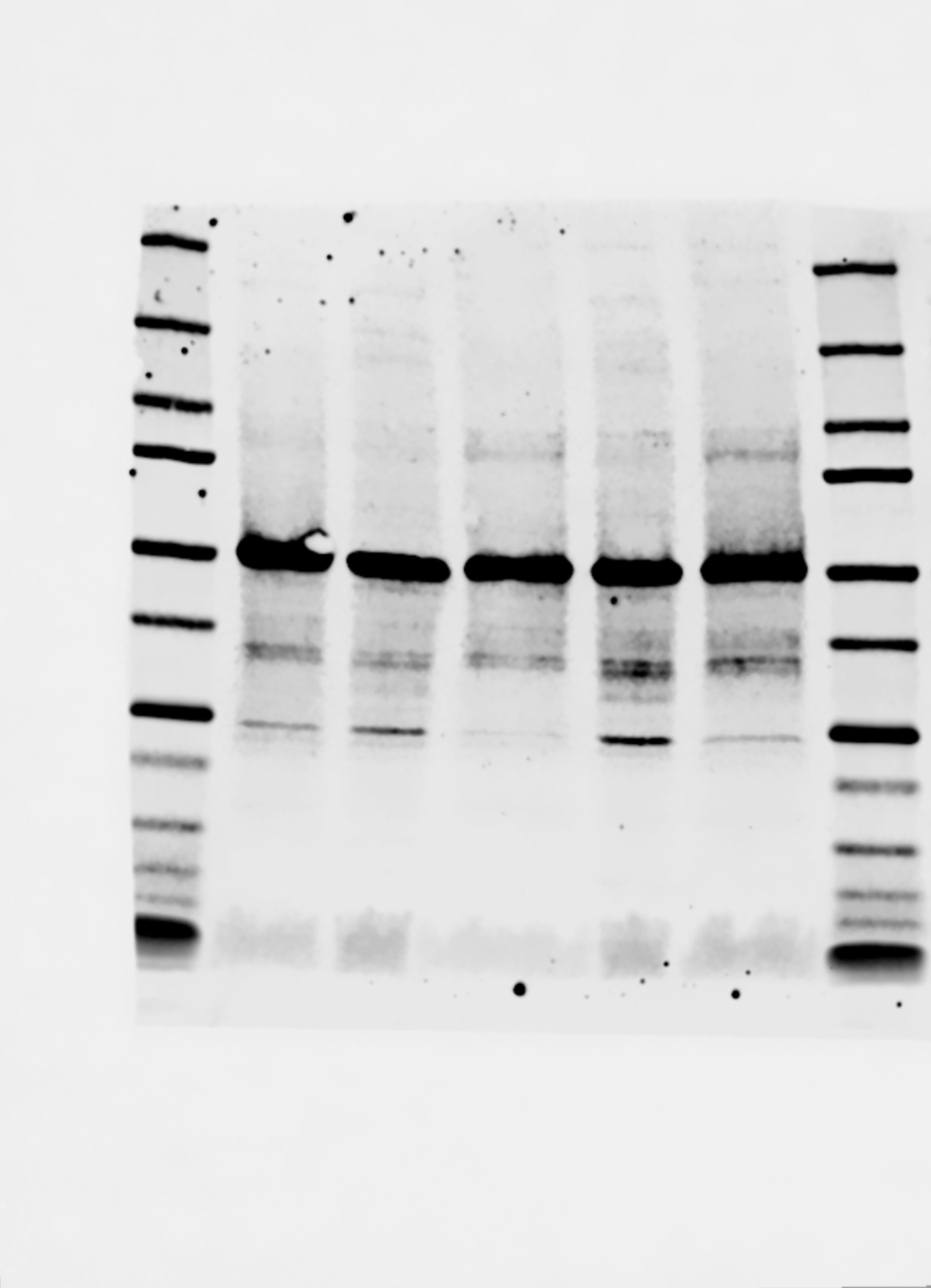
**

**
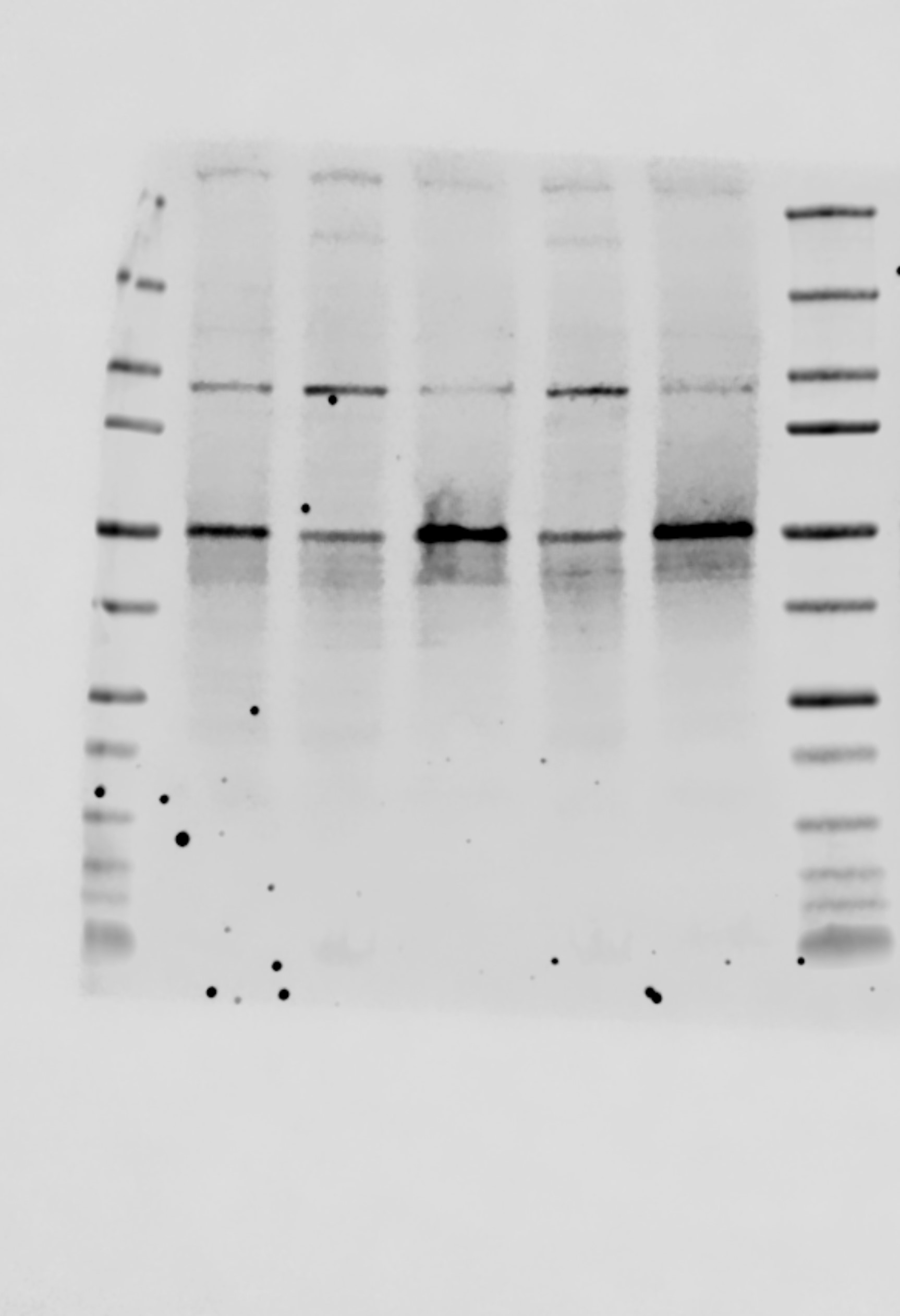
**

**Figure S7. Uncropped membranes of the western blot for alpha tubulin, MDM4 and p53 shown in figure 3C.**

**Figure S8. A Competition assay in embryoid bodies and monolayer culture during 4 days of spontaneous and neuroectoderm directed differentiation.**

**A** Embryoid bodies spontaneously differentiated for 4 days were generated with hESC lines VUB03^wt^, VUB19^wt^, VUB03^1q32^ and VUB19^1q21.1^. In the siMDM4 condition, the hESC^1q^ have been treated with siRNA against *MDM4* prior to mixing.

**B** Embryoid bodies during 4 days of directed neuroectoderm differentiation. Setup is similar to panel A.

**C** Competition assay during spontaneous differentiation in monolayer culture. Mixes were created with hESC lines VUB03^wt^, VUB19^wt^, VUB03^1q32^, VUB03^1q21.^1 and VUB19^1q21.1^.

**D** Quantification of 1q-cells at the start of the monolayer cell competition during spontaneous differentiation (day 0) and at day 4, with and without siRNA against MDM4.

**E** mRNA expression of differentiation markers EOMES and MIXL1 and pluripotency marker NANOG after 4 days of monolayer culture from panel C. Dotted line represents expression in hESC.

**F** mRNA expression of differentiation markers PAX6 and OTX2 and pluripotency marker NANOG after 4 days of embryoid bodies from panel B. Dotted line represents expression in hESC.

**G** mRNA expression of differentiation markers PAX6, OTX2, SOX17, TBXT and GATA4 and pluripotency markers NANOG and POU5F1 after 4 days of spontaneously differentiated embryoid bodies from panel A. Dotted line represents expression in hESC.
