# Supplementary figures and images for: Gain of 1q confers an MDM4-driven growth advantage to undifferentiated and differentiating hESC while altering their differentiation capacity
Source: Cell Death Dis. 2024 Nov 21;15(11):852. doi: 10.1038/s41419-024-07236-x (PMC11582570; doi:10.1038/s41419-024-07236-x)

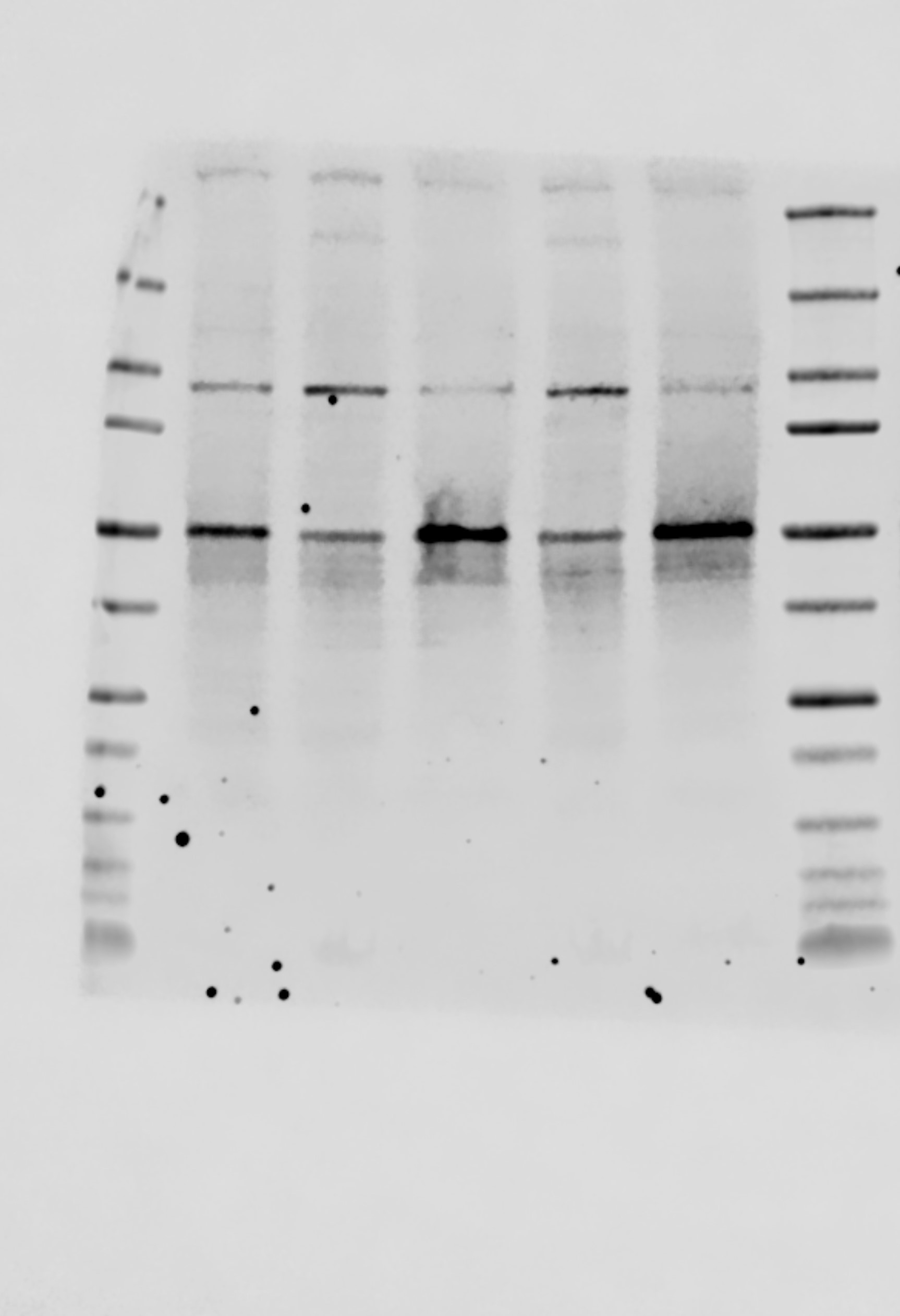

Supplement: Supplementary file 8 — Uncropped Westernblot Figure 3C [file 41419_2024_7236_MOESM8_ESM.jpg]

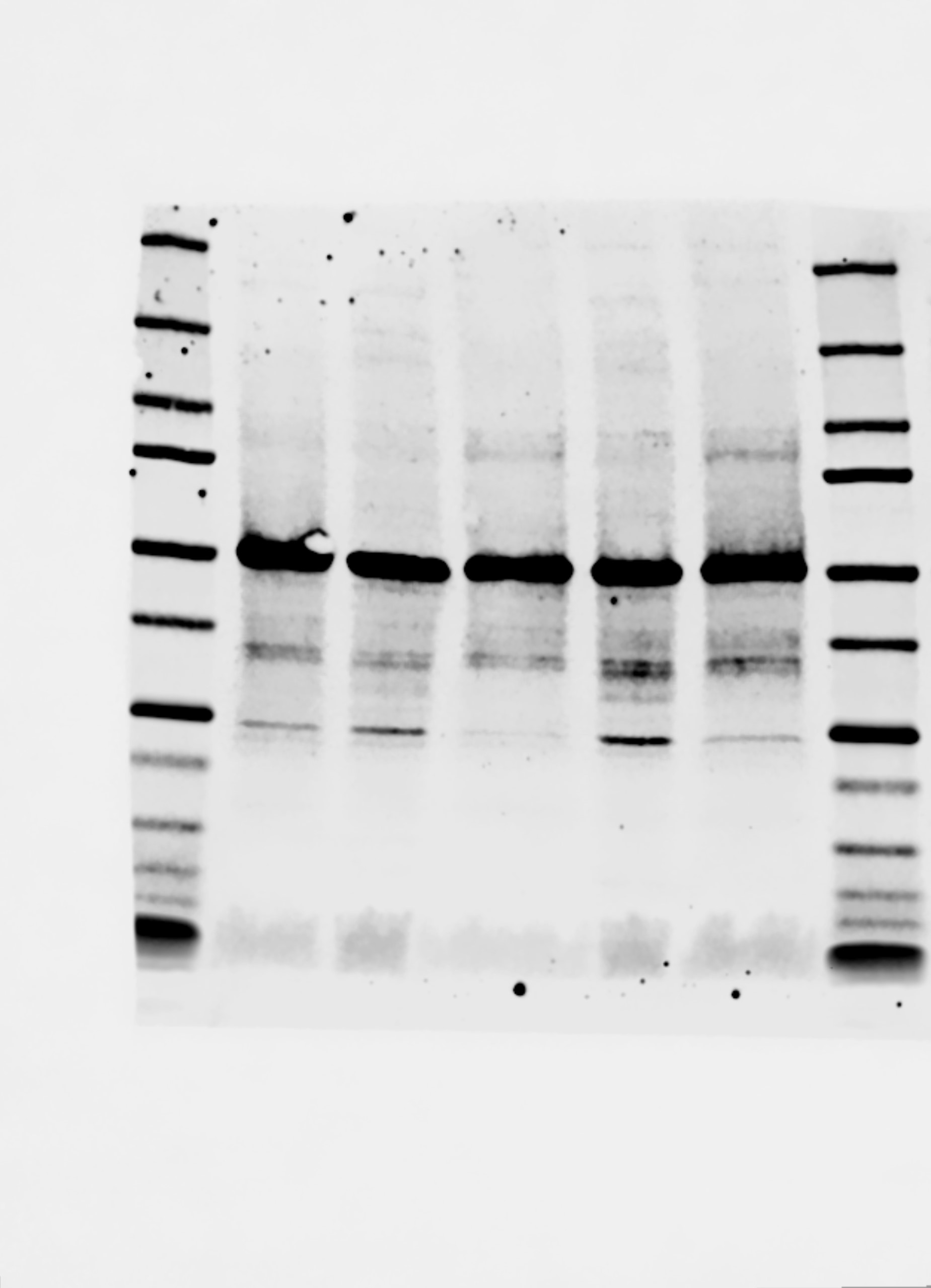

Supplement: Supplementary file 9 — Uncropped Westernblot Figure 3C [file 41419_2024_7236_MOESM9_ESM.jpg]
